# Passive Acoustic Data as Phenological Distributions: Uncovering Signals of Temporal Ecology

**DOI:** 10.1002/ece3.73020

**Published:** 2026-02-03

**Authors:** Mary K. Clapp, Morgan W. Tingley, Damon B. Lesmeister, Scott A. Gremel, Jason I. Ransom, Mandy L. Holmgren, Rodney B. Siegel

**Affiliations:** ^1^ The Institute for Bird Populations Petaluma California USA; ^2^ Ecology and Evolutionary Biology University of California—Los Angeles Los Angeles California USA; ^3^ USDA Forest Service Pacific Northwest Research Station Corvallis Oregon USA; ^4^ National Park Service Sedro‐Woolley WA USA

**Keywords:** acoustic data, hierarchical generalized additive models, Olympic National Park, passive acoustic data, phenology, vocal activity

## Abstract

Passive Acoustic Monitoring (PAM) is an increasingly common method for monitoring birds and other sound‐producing organisms at scale, but methods that digest these data streams into ecological insight remain underdeveloped. Specifically, using PAM and classification algorithms powered by artificial intelligence (AI) to uncover the phenology of vocal animals is an emerging use of these data but currently lacks standardized, repeatable methods with verified connections to biological phenomena. Here, we articulate specific hypotheses regarding the relationship between avian vocal activity and phenological events, and present a flexible, reproducible methodological pipeline for quantifying avian vocal phenology from PAM data. We applied our pipeline to 18,568 h of audio from 185 recording sites across Olympic National Park, USA. We processed acoustic data through an AI species classifier (BirdNET), then filtered the output using species‐specific precision thresholds established through expert review to minimize false positives. For 25 species representing diverse migratory strategies across two elevational strata, we used hierarchical generalized additive models (HGAMs) to estimate daily probabilities of vocal activity from which we extracted standardized “phenometrics” describing the timing, duration, and shape of vocal activity curves. PAM‐derived patterns of phenometrics broadly supported expectations, showing promise for future expansion of these methods. Resident species generally exhibited earlier and longer vocal periods than migratory species, and birds at mid‐elevations showed delayed and shortened vocal phenology relative to lower elevations. Many species displayed bimodal vocal patterns, with secondary peaks 30–50 days after initial peaks. These generalizable patterns of vocal phenology likely cue transitions in various stages of the avian breeding cycle. Late‐season vocal activity, especially in irruptive and resident species, highlighted the method's capacity to capture ecological transitions beyond the breeding season, but robust inferences require further ground‐truthing. This study advances the use of PAM for phenological research, offering outputs that can inform long‐term monitoring and detect phenological shifts in response to environmental change. We make recommendations for methodological and technological advancement, and highlight the need for studies that integrate PAM data and field‐based observation to further strengthen the links between observed phenometrics and confirmed biological states of vocal organisms.

## Graphical abstract

Passive acoustic monitoring has the potential to broaden our understanding of vocal animals' annual phenological cycles throughout and beyond the breeding season. We present standardized analytical methods for quantifying vocal phenology from passive acoustic recordings processed by classification algorithms, guided by clearly articulated hypotheses about vocal activity and phenological events.

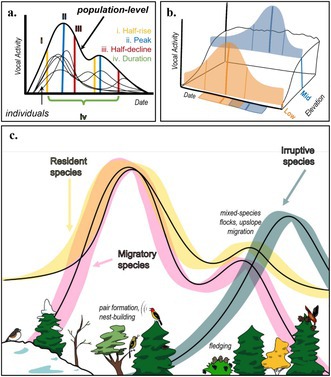

## 1 Introduction

The vast proliferation of passively collected acoustic data over the past decade has allowed researchers to collect information about vocal animals at an unprecedented pace and scale (Sugai et al. 2019). This increase in the prevalence of passive acoustic monitoring (PAM) has been facilitated by technological innovation in the hardware used to collect and store the data, the computing power needed to process it, and the software used to analyze it. Notably, the development and application of artificial intelligence (AI) to process the resulting datasets—often at the scale of terabytes to petabytes—has widened a crucial bottleneck in the PAM process from data collection to biological inference (Sethi et al. 2024). Analytical advances in this process have focused primarily on improving the classification of species using AI models (Kahl et al. 2021; Lapp et al. 2023; Pérez‐Granados 2023) and defining appropriate ecological models for determining site occupancy and abundance from acoustic data (Cole et al. 2022; Rhinehart et al. 2022; Navine, Camp, et al. 2024). Increasingly, attention is focusing on bridging the fields of population biology and behavioral ecology, connecting landscape‐scale patterns in PAM‐derived data with behavior at the level of species and individuals (Kitzes et al. 2021; McGinn et al. 2023; Knight et al. 2024), and imagining acoustic recordings as a trove of untapped ecological signal.

Of the various potential signals contained within bioacoustics—including occupancy, abundance, behavior, and demography—acoustic records are particularly well‐suited to describing the phenology of vocal species throughout their annual cycle (Oestreich et al. 2024). Across taxa, shifts in phenology—in the timing of breeding, migration, and other critical life history events—have been widely documented in response to climate change (Thackeray et al. 2016). Early investigations of phenology using PAM employed acoustic indices as abstract proxies for species‐ or community‐level “biophony” (Pijanowski et al. 2011), circumventing the need to identify individual sounds that comprise the biophony, under the assumption that vocal behavior is a proxy for phenological events such as the arrivals and departures of migratory birds or the onset of breeding condition (Oliver et al. 2018; Buxton et al. 2018). Applying AI classification to acoustic data builds on this theoretical framework and yields species‐ (or finer) level classifications that potentially inform how phenological distributions embedded in bird vocalizations are generalizable to, or differ among, species, populations, and individuals, including variation by categories like functional group, migratory strategy, region, age, or sex. The greater specificity of AI‐classified sounds also makes it possible to directly test hypothesized correlations between vocal activity and particular behavioral states or phenophases with field‐collected observations or validated data, which is key to interpreting acoustic data streams at scale.

**FIGURE 1:**
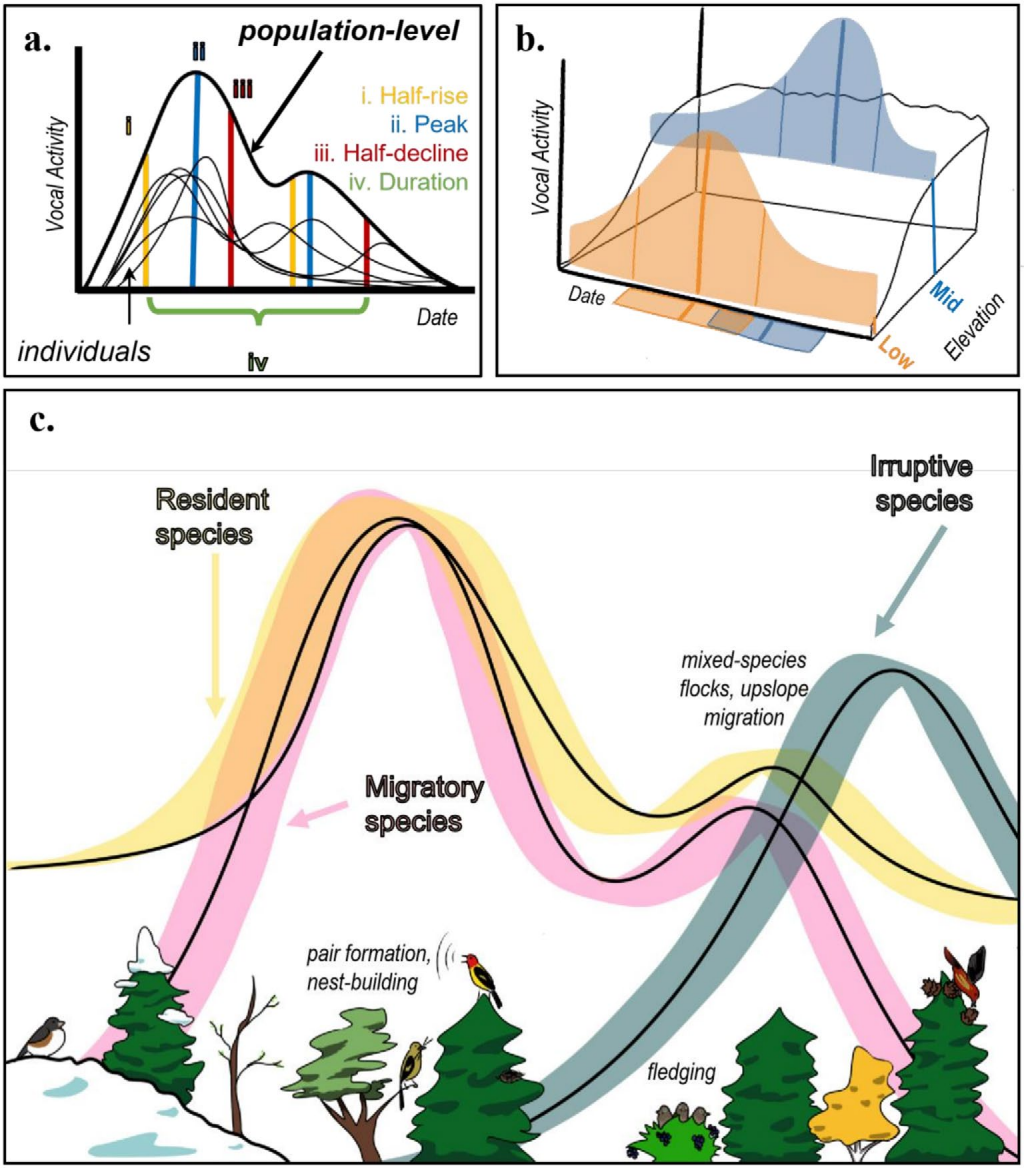
Schematic diagram of how avian vocal activity may relate to phenological events. (a) The vocal activity of individual birds of a species in a region can be summarized by a cumulative function or mean, which represents population‐level vocal phenology, and variance that represents individual variation in individual activity. Metrics of the population‐level phenological distribution, “phenometrics”, can be calculated and compared across species, regions, or years. (b) Phenological distributions can also be evaluated along environmental gradients, such as elevation. (c) Species‐level distributions can be further aggregated to and compared across functional groups or communities.

PAM provides opportunities to study phenological shifts across the entire span during which a life‐history event occurs within a population—to quantify, summarize, and compare phenological phenomena in terms of continuous processes or “phenological distributions”—rather than as a single measurement such as date of first flower or first egg laid (Inouye et al. 2019). Because acoustic recorders can be readily deployed for months at a time and collect effectively continuous data, resulting data on full phenological distributions enable testing hypotheses that are key to understanding the phenological responses of individuals, populations, and species to environmental change (Figure 1). For instance, continuous acoustic data could be used to capture the entire duration or variance of a phenophase, such as egg‐laying, at the population level, or to predict phenological overlap between two interacting species as overlap in the integrals of two curves.

Studying temporal patterns in the vocalization frequency of birds may also spark new ecological and natural history insight (Tosa et al. 2021; Ross et al. 2023; Kitzes et al. 2025). For example, early ornithological studies of oscine passerines have long provided evidence for a link between the singing frequency of breeding male birds and phases of the breeding cycle in temperate regions (Slagsvold 1977; Greig‐Smith 1982; Lampe and Espmark 1987) as male songbirds defend territories, attract mates, and maintain pair bonds; their singing rates peak at the onset of breeding and are followed by a non‐linear decline. This peak singing rate is often associated with peak detectability in point count surveys (Strebel et al. 2014; Furnas and McGrann 2018)—which has been used as a metric for studying phenological shifts over many decades (Socolar et al. 2017). But passive acoustic data—with its intensive sampling over near‐continuous time—has the potential to provide much more nuanced quantitative tracking of vocal detectability than identifying a single peak. Indeed, vocal activity extends beyond breeding‐associated song, encompassing fledging and post‐fledging dispersal, social flocking behavior, molt, and molt‐migration (Hahn et al. 2015; Gilbert 2022), and beyond the geographically biased paradigm of male birdsong, encompassing female song and non‐breeding song (Odom et al. 2014; Rose et al. 2022; Wu et al. 2025). Thus, PAM has great potential to broaden our understanding of birds' habitat use and phenological cycles throughout and beyond the breeding season (Figure 1c), if methodological hurdles involving soundscape recording, data processing, and analysis can be overcome. Without standardized methods guided by clearly articulated hypotheses about vocal activity and phenological events, much of the potential information embedded in PAM datasets remains underutilized.

Here, we present a flexible, reproducible workflow for modeling avian vocalization frequency over continuous time and extracting potentially relevant phenophases. We illustrate this process with birds, using BirdNET to automate avian species identification within recordings, and perform validation to minimize false positive detections. We then fit hierarchical generalized additive models (HGAMs) to derive several vocal phenometrics that describe the shape, extent, and duration of vocal activity. We apply this methodology to an acoustic dataset of 18,568 audio‐hours recorded across Olympic National Park, Washington, USA, and describe vocal phenology for 25 diurnal bird species that vary in migratory strategy and elevational range. This workflow is designed for flexibility, as it can accommodate a variety of classifiers, scale to large datasets, and is applicable to any sound‐producing taxa with seasonal patterns.

Using the derived phenometrics, we test multiple hypotheses about vocal activity in birds: (1) vocal frequency over the course of a temperate‐breeding bird's summer season is unimodal with respect to time, with peak vocal activity corresponding largely to early breeding season; (2) resident species' vocal activity curves will be identifiable by non‐zero probabilities of vocal activity in the pre‐breeding season, whereas migratory species will exhibit narrower windows of vocal activity within the annual cycle, bracketed by their arrival and departure; and (3) vocal phenology will shift with elevation, with later onset and shorter duration at higher elevations. These hypotheses are broadly supported by the avian ecology literature (Perrins 1970; Hahn et al. 2015; Boyle et al. 2016), so demonstrating them with acoustic data would provide proof‐of‐concept that PAM can offer robust inference on avian phenology. As acoustic datasets continue to grow globally, our approach offers a scalable solution for unlocking the ecological potential of PAM and applying it to pressing questions in temporal ecology and biodiversity monitoring.

## 2 Methods

### 2.1 Field Methods

In 2021, we deployed autonomous recording units (ARUs; SM4s, Wildlife Acoustics, Maynard, PA) across Olympic National Park, Washington, USA, to monitor avian vocal activity along environmental gradients during the breeding season (April—September). Although ARUs were originally deployed as part of a broader project targeting *Strix* owls, these devices simultaneously recorded diurnal songbird vocalizations, providing a rich dataset for phenological analysis. Four ARUs were distributed within 5‐km^2^ hexagons, spaced at least 500 m apart to minimize spatial autocorrelation (Lesmeister and Jenkins 2022). Hexagon selection followed a stratified random sampling design to capture variation across elevational gradients and habitat types within forested land. Forest composition was primarily dominated by Douglas‐fir ( *Pseudotsuga menziesii* ), Sitka spruce ( *Picea sitchensis* ), and western hemlock ( *Tsuga heterophylla* ). We defined two elevation strata on the basis of U.S. National Park Service Inventory and Monitoring protocols for Olympic National Park: “Low” (< 650 m elevation, *n* = 64 sites) and “Mid” (650–1350 m elevation, *n* = 121 sites).

Recorders were placed 2 m from the ground on small‐diameter trees (15–20 cm at recorder height) to minimize physical obstruction to the microphones. We recorded at a quality of 16‐bit WAV with a sampling rate of 32 kHz and gain of 16 dB using an external omnidirectional microphone standard to SM4 ARUs. To standardize recording quality, microphones were no older than 3 years and were tested and calibrated prior to field deployment using an Extech 407,766 sound calibrator (Industrial Electronics Inc., Knoxville, TN) and Wildlife Acoustics' utility (Wildlife Acoustics 2024). ARUs recorded in two crepuscular blocks for 2 h prior to and after local sunset and sunrise, as well as an additional 10 min on each hour. For this analysis, we analyzed the morning recordings taken 2 h following sunrise plus two additional 10‐min samples at 0800 and 0900 h, totaling approximately 140 min of recording per day per site. Deployment duration averaged 42 ± 14 days per site; exact windows of deployment varied by recorder depending on seasonal accessibility (Figure S1).

### 2.2 Acoustic Data Processing

We processed raw acoustic data through the global version of BirdNET 2.4, a convolutional neural network trained on globally sourced bird sounds that produces predictive labels of bird species in acoustic recordings (Kahl et al. 2021). We processed nearly 10 million consecutive, non‐overlapping 3‐s windows (i.e., the algorithm attempts a species prediction for every 3‐s sample of audio), left the sensitivity setting at its default of 1, and set the minimum Confidence Score to 0.1 to maximize recall on the initial run.

BirdNET returns as output a table of species labels and their corresponding “Confidence Score” (CS), a measure between 0 and 1, which roughly approximates how good a match the sample is to the examples of that species that the model was trained on. These labels are predictions, not definitive identifications or “detections,” and are subject to false positives, which must be accounted for prior to analysis, by filtering out labels below a certain CS, during analysis, by modeling a false positive rate (Chambert et al. 2018; Spiers et al. 2022; Rhinehart et al. 2022), or both. We chose the former thresholding approach, implementing a species‐specific verification protocol to account for differences in model precision across species (Wood and Kahl 2024). Similar to human‐collected data (e.g., point counts), classifier outputs are also subject to false negatives, or failures to detect a species when it is present. We did not explicitly measure recall in this study, since we prioritized minimizing the incidence of false positives.

We selected 29 diurnal bird species commonly detected in regional avifaunal surveys (Siegel et al. 2012) for expert verification and modeling. These species vary by taxonomic order, migratory strategy (i.e., resident, short‐range migrant, long‐range migrant), and relative abundance within two elevation strata (Table 1). This selection allowed us to evaluate how vocal phenology varies with ecological traits and environmental context.

**TABLE 1:** We attempted to model vocal phenology for species (listed alphabetically by species code within each migratory strategy group) in low and mid‐elevation strata if their published elevation ranges (modeled range containing 95% of observations from 2002 to 2003 point count surveys) at Olympic NP overlapped the stratum (Siegel et al. 2012).

Species code	Common name (scientific name)	Migratory strategy	Mean (SD) elevation (m)	Range
BRCR	Brown Creeper ( *Certhia americana* )	R	475 (431)	17–1394
CAJA	Canada Jay ( *Perisoreus canadensis* )	R	1119 (502)	39–1730
CBCH	Chestnut‐backed Chickadee ( *Parus rufescens* )	R	533 (491)	8–1602
GCKI	Golden‐crowned Kinglet ( *Regulus satrapa* )	R	708 (597)	9–1702
NOFL	Northern Flicker ( *Colaptes auratus* )	R	1232 (639)	77–1913
PAWR	Pacific Wren ( *Troglodytes pacificus* )	R	542 (531)	6–1585
PISI	Pine Siskin ( *Carduelis pinus* )	R	1470 (394)	100–1923
PIWO	Pileated Woodpecker ( *Dryocopus pileatus* )	R	202 (144)	35–514
RBNU	Red‐breasted Nuthatch ( *Sitta canadensis* )	R	1148 (562)	66–1875
SOGR	Sooty Grouse (*Dendragapus fuliginosus*)	R	846 (596)	73–1875
STJA	Steller's Jay ( *Cyanocitta stelleri* )	R	200 (279)	6–1061
AMRO	American Robin ( *Turdus migratorius* )	SDM	378 (536)	5–1873
DEJU	Dark‐eyed Junco ( *Junco hyemalis* )	SDM	1192 (564)	64–1908
HETH	Hermit Thrush ( *Catharus guttatus* )	SDM	1352 (334)	567–1889
VATH	Varied Thrush ( *Ixoreus naevius* )	SDM	749 (575)	7–1670
BTYW	Black‐throated Gray Warbler ( *Dendroica nigrescens* )	LDM	159 (201)	4–846
HAFL	Hammond's Flycatcher ( *Empidonax hammondii* )	LDM	442 (365)	58–1331
OSFL	Olive‐sided Flycatcher ( *Contopus borealis* )	LDM	1293 (470)	224–1893
RUHU	Rufous Hummingbird ( *Selasphorus rufus* )	LDM	654 (645)	9–1694
SWTH	Swainson's Thrush ( *Catharus ustulatus* )	LDM	139 (185)	3–680
TOWA	Townsend's Warbler ( *Dendroica townsendi* )	LDM	457 (352)	43–1602
WAVI	Warbling Vireo ( *Vireo gilvus* )	LDM	293 (319)	4–1169
WEFL	Western Flycatcher ( *Empidonax difficilis* )	LDM	336 (340)	6–1190
WETA	Western Tanager ( *Piranga ludoviciana* )	LDM	227 (178)	27–647
WEWP	Western Wood‐Pewee ( *Contopus sordidulus* )	LDM	29	n/a
WIWA	Wilson's Warbler ( *Wilsonia pusilla* )	LDM	186 (262)	3–934
YRWA	Yellow‐rumped Warbler ( *Dendroica coronata* )	LDM	1545 (476)	141–1897
EVGR	Evening Grosbeak ( *Coccothraustes vespertinus* )	IRR	963 (664)	279–1514
RECR	Red Crossbill ( *Loxia curvirostra* )	IRR	897 (649)	30–1858

*Note:* Key for migratory strategies: *R* = resident (yellow), SDM = short‐distance migrant (pink), LDM = long‐distance migrant (blue), IRR = Irruptive (green).

**FIGURE 2:**
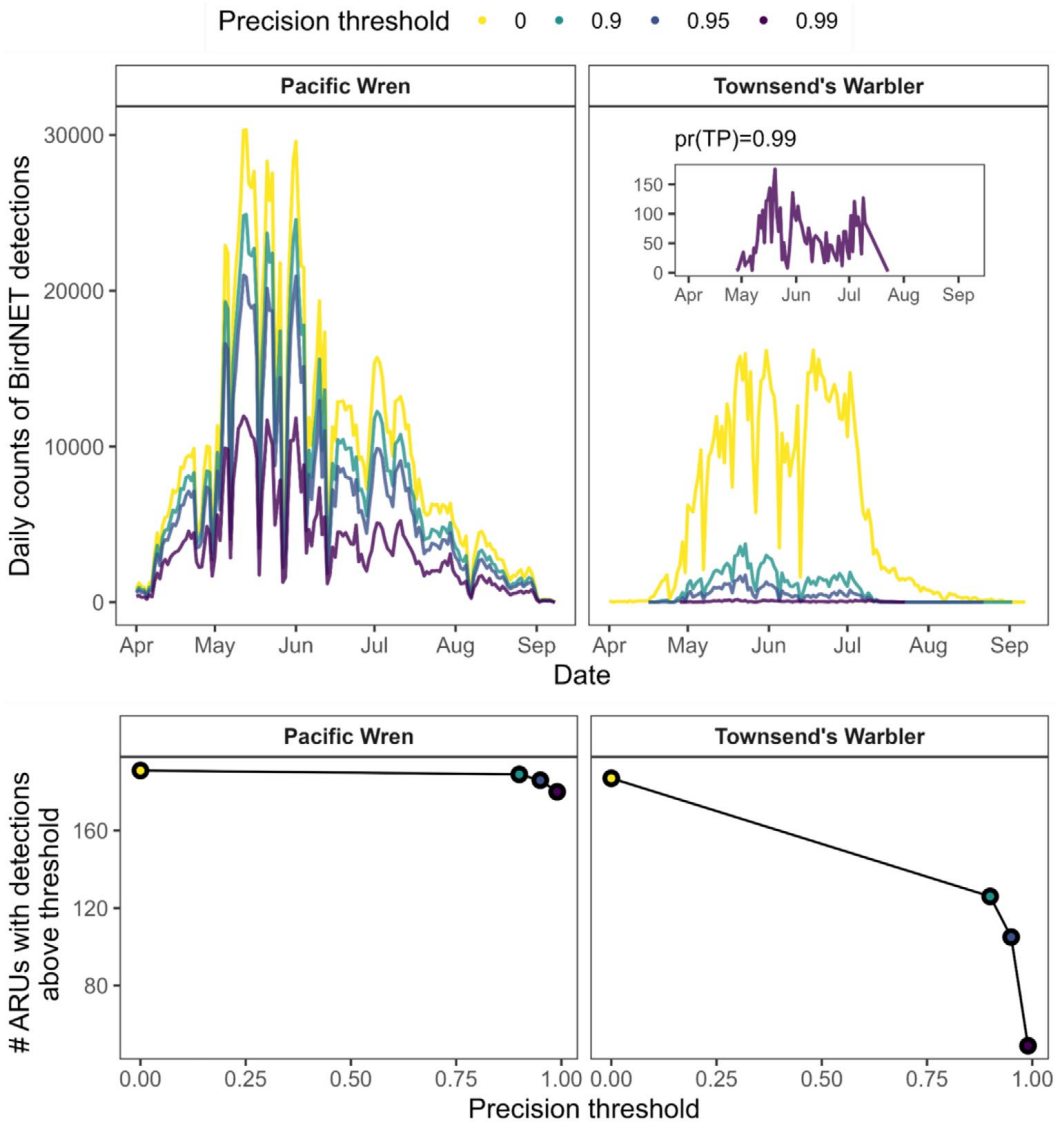
Illustration of how classifier precision threshold can influence effective sample size and phenological patterns in acoustic data. (Top) Counts of BirdNET detections for two common species at Olympic National Park, thresholded by different true‐positive probabilities. (Bottom) As the true‐positive threshold becomes more stringent and more BirdNET predictions are filtered out of the dataset, the number of recording locations with BirdNET detections decreases for Townsend's Warbler.

For each species, a reviewer with expertise in aural bird identification examined randomly selected BirdNET labels (3‐s clips) across two CS ranges (0.1–1.0 and 0.95–1.0), for 200 clips total. The observer assigned a 0 to the clip if the focal species was absent, and a 1 if it was present, and additionally applied a vocal class label to the sample (Pieplow 2019). Samples whose ID could not be confidently determined were categorized as “0” to safeguard the validation set against possible false positives. We used the binary manual validation outcomes to fit logistic regression models for each species, predicting the probability of a true positive, or pr(TP), as a function of a sample's CS. Using the results of those regressions, we calculated species‐specific precision thresholds corresponding to pr(TP) ≥ 0.95 and filtered out all BirdNET samples with a CS below the calculated threshold. To illustrate sensitivity to this filtering step, we conducted case studies on two common species: Pacific Wren ( *Troglodytes pacificus* ) and Townsend's Warbler (*Setophaga townsendii*), investigating the extent to which different precision thresholds altered daily counts of BirdNET labels, the total number of recording locations with labels, and estimates of phenometrics (Figure 2).

We tolerated a pr(TP) under 1 because, unlike in occupancy models, where a single false positive can “flip” site‐level estimates from unoccupied to occupied and severely bias estimation (McClintock et al. 2010), our analysis aimed to estimate call density, wherein occasional false positives amid hundreds to thousands of true positive detections have less of an impact on inference (assuming they are randomly distributed). Because thresholding also filters out true positive instances (sometimes hundreds to thousands), the vocalization counts represent undercounts of true vocal activity and are relative indices. Stringent thresholding has been shown to functionally correspond to reduced effective sampling area of the ARU (Knight and Bayne 2019); however, we accepted this possibility as a tolerable trade‐off for high precision.

Classifier performance could vary seasonally if bird species' vocalizations vary over the course of the season to the point that the classifier applied to the data is appreciably worse at detecting or correctly labeling those sounds. This could arise by birds singing less crystallized songs early or late in the season, by differences in relative output of sound classes (e.g., songs, calls, drums) across the season (Figures S3 and S4), by environmental differences impacting sound transmission (e.g., leaf‐out), and likely many more factors. It is well established that these biological phenomena vary seasonally (Wiley and Richards 1978; Best 1981; Blumenrath and Dabelsteen 2004), but it is generally unknown how classifier performance varies with them. Such “distribution shifts” could bias ecological analyses if they are unaccounted for (Navine, Denton, et al. 2024; van Merriënboer et al. 2024). We assessed our existing annotations for signatures of bias by elevation and season using a model comparison approach in the logistic regression where pr(TP) is estimated. We compared a “base” model (score as the only predictor) with models that also include ordinal day of year and elevation stratum, respectively, as predictors. We compared model fit using Akaike's Information Criterion (AIC) to assess whether models containing environmental covariates explained more variation than our base (score‐only) model.

### 2.3 Quantifying Vocal Phenology

Once data were thresholded by species, we quantified vocal activity as a daily “success rate” for each species at each site, defined as the number of BirdNET IDs for that day divided by the total number of analyzed 3‐s segments for that day. This binomially distributed ratio intrinsically accounts for variation in sampling effort over days and sites, which is common in PAM data, and is similar to the “call density” metric described in other recent work (Navine, Denton, et al. 2024).

We used hierarchical generalized additive models (HGAMs) to model vocal activity for each species in each of two elevational strata. GAMs are useful in modeling complex, non‐linear or “wiggly” patterns such as time‐series data (Heit et al. 2024), whereas HGAMs allow for the predictive surface of the GAM to vary by levels of a grouping variable (Pedersen et al. 2019). We fit the HGAMs using the ‘mgcv’package in R (Wood 2017). Each model treated the daily success rate as a binomial response, with a thin‐plate regression spline for ordinal day (k = 7, bs = “tp”) and a random intercept for ARU site to account for spatial autocorrelation between measures within sites. Although we initially experimented with cyclic splines (bs = “cc”) to model year‐round phenology, they were inappropriate for this seasonally constrained dataset. Datasets with full annual coverage may be better represented by cyclic splines.

We attempted to fit HGAMs to any species‐by‐elevation stratum combination for which 5 or more ARU locations had 1 or more BirdNET predictions above our species‐specific threshold (Table 2) for any day of recording. We made no assumptions about the breeding occupancy status of birds at each ARU location and assumed that birds moved over the course of the recording season. We also assumed the number of individuals per species captured by each ARU is a latent quantity that varies with respect to time, microhabitat suitability, positions of individual territories, and other factors. Thus, the models we fit represent a population‐level phenological distribution of vocal activity over the migration and breeding seasons; an emergent property of many individuals' vocal outputs, which is inclusive of, but not limited to, the vocal activity of birds on occupied breeding territories. Adaptations of this method, which endeavor to estimate vocal activity of individuals or at specific recording locations (e.g., as for occupancy or density analyses) would need to calibrate vocal activity with additional data on the number of individuals present at the site.

**TABLE 2:** Summary of acoustic data processing method for 29 bird species (listed alphabetically by species code) in two elevation strata at Olympic National Park for which manual verification of BirdNET data was performed, including: Calculation of a precision threshold, fitting of a hierarchical GAM on the basis of elevation range overlap, and detection of a vocal phenoperiod.

Species Code	CS @ pr(TP) = 0.95	Total number of BirdNET hits	Number of BirdNET hits above threshold	Fraction BirdNET hits retained	Low elevation			Mid elevation		
					Model attempted (range overlap)	Model fit (sufficient data)	Phenoperiod detected	Model attempted (range overlap)	Model fit (sufficient data)	Phenoperiod detected
AMRO	0.146	16,677	10,743	0.64	1	1	1	1	1	1
BRCR	0.85	509,399	20,201	0.04	1	1	1	1	1	1
BTYW	1	220,160	0	0.00	0	0	0	0	0	0
CAJA	0.216	10,840	5800	0.54	1	1	1	1	1	1
CBCH	0.335	710,315	359,933	0.51	1	1	1	1	1	1
DEJU	0.116	241,368	217,738	0.90	1	1	1	1	1	1
EVGR	0.18	20,948	17,792	0.85	1	1	1	1	1	1
GCKI	0.846	1,401,440	68,651	0.05	1	1	1	1	1	1
HAFL	0.571	107,416	44,027	0.41	1	1	1	1	1	1
HETH	0.277	57,210	32,260	0.56	0	0	0	1	1	1
NOFL	0.226	24,629	17,504	0.71	1	1	0	1	1	1
OSFL	0.243	44,227	34,151	0.77	1	1	1	1	1	1
PAWR	0.42	1,524,842	1,027,812	0.67	1	1	1	1	1	1
PISI	0.089	61,851	61,851	1.00	1	1	1	1	1	1
PIWO	0.624	19,764	7223	0.37	1	1	1	0	0	0
RBNU	0.022	660,600	660,600	1.00	1	1	0	1	1	1
RECR	0.107	533,624	517,032	0.97	1	1	1	1	1	1
RUHU	0.995	2945	6	0.00	1	0	0	1	0	0
SOGR	0.462	63,505	15,691	0.25	1	1	1	1	1	1
STJA	0.221	92,441	69,439	0.75	1	1	1	1	1	0
SWTH	0.256	8681	4848	0.56	1	0	0	0	0	0
TOWA	0.872	818,191	46,607	0.06	1	1	1	1	1	1
VATH	0.462	944,650	439,384	0.47	1	1	1	1	1	1
WAVI	0.492	10,363	2942	0.28	1	1	1	1	0	0
WEFL	0.154	1,528,095	1,323,174	0.87	1	1	1	1	1	1
WETA	0.188	40,498	26,318	0.65	1	1	1	1	1	1
WEWP	0.53	2409	809	0.34	1	0	0	0	0	0
WIWA	0.675	55,494	8046	0.14	1	1	1	0	0	0
YRWA	0.548	16,090	2150	0.13	1	0	0	1	1	1
Total		9,748,672	5,042,732	0.52	27	23	21	24	22	21

*Note:* Species codes defined in Table 1. “CS” = BirdNET Confidence Score. Grey shading emphasizes the point at which data for the species was insufficient to achieve the step in the methods defined by the columns.

We used each HGAM to predict vocalization probabilities for each species‐by‐elevation combination over a date range that matched the PAM data collection efforts using the ‘predict()’ function. From the fitted values, we extracted the following phenometrics (Figure 1a): (1) peak(s) in detectability, defined as the day any maximum value of predicted vocal probability exceeded 30% of the model's highest maximum; (2) half‐rise(s), the day at which the predicted probability of vocalization was halfway between a local minimum and the next chronological local maximum; and (3) half‐decline(s), or the day at which the predicted probability of vocalization was halfway between a local maximum and the next chronological local minimum. We further assumed that each species has a defined vocalization phenoperiod, during which it is both present and vocally detectable within a breeding season. Thus, we used the difference between the (first) half‐rise and the (final) half‐decline to summarize (4) seasonal duration of the phenoperiod. When, at the start and/or end of the modeled seasonal period, the probability of vocalization was predicted to be within at least 30% of the seasonal maximum, we used the first or last date of the monitoring period as the start and/or end of the phenoperiod, respectively. These phenometrics enabled standardized cross‐species and cross‐stratum comparisons of vocal phenology.

## 3 Results

### 3.1 Acoustic Data Processing

Verifying 200 samples for each of 29 species required approximately 160 observer hours, or 5.5 h per species. The relative distribution of vocal classes (songs, calls, etc.) within the 200‐sample verification sets varied widely by species, but in most cases, for birds with song, the majority of samples belonged to the “song” class (Table S1).

The predicted minimum Confidence Score (CS) corresponding to a true positive rate of at least 95% varied widely by species (Table 2). Thresholding the data reduced the total number of BirdNET IDs by roughly half, though removal rates varied substantially by species. For example, filtering Pacific Wren data retained 67% of total labels, whereas Townsend's Warbler retained only 6%.

Exploring the impact of thresholding on the BirdNET output of Pacific Wren and Townsend's Warbler depicted the consequences of using increasingly stringent values of pr(TP) (Figure 2). For the wren, whose 95% precision threshold was CS = 0.41, both the number of sites with labels as well as phenometric estimates were robust to thresholding except for at the 0.99 threshold, where no phenoperiod was estimated from the remaining data (Figure 2, Figure S2). For the warbler, whose 95% precision threshold was CS = 0.87, thresholding led to increasingly steep reductions in the number of locations that contained any labels for that species (Figure 2). At the most stringent threshold (0.99), seasonal patterns in vocal activity were retained, but phenometric estimates shifted by several days (Figure S2).

We assessed our verification method post hoc for evidence of classifier performance shifts over time and/or space by comparing our base model explaining pr(TP) with only “score” as a predictor to models also including ordinal day of year and elevation stratum, respectively. On the basis of AIC model comparison, our base model was better ranked than a model with elevation for 89% of species (16/18) whose vocal activity was modeled in both elevation strata (Table S3). Our base model was also better ranked than a model with ordinal day of year for 72% of species (21/29; Table S3), indicating that a minority of species may have raw phenological signals in vocalization biased by seasonal changes in classifier efficacy. Predicted pr(TP) for those species fell below 0.95 for extreme values of day of year (Figures S5 and S6). When the 95% threshold was applied to the validation datasets, it greatly reduced or eliminated most of the false positive samples that drove the relationships with day of year and elevation stratum (Figures S7 and S8), indicating that controlling false positives by thresholding may be sufficient for estimating unbiased relative indices of vocalization intensity across the environmental covariates important to this study.

### 3.2 Pipeline and Model Performance

Our analytical pipeline processed over 10 million three‐second audio segments collected across 185 ARU deployments. BirdNET identified millions of initial candidate labels, which, following expert‐informed filtering, yielded sufficient data for modeling 25 of the 29 species on which verification was performed; 22 and 23 species in Low and Mid elevation strata, respectively (Table 2). Modeling performance varied by species and stratum. Most species‐elevation models exhibited deviance explained values exceeding 40% (Table S2). Models for the Mid and Low elevation strata had similar average deviance explained values (Low, 0.588; Mid, 0.604; Table S2).

### 3.3 Phenological Patterns

**FIGURE A1:**
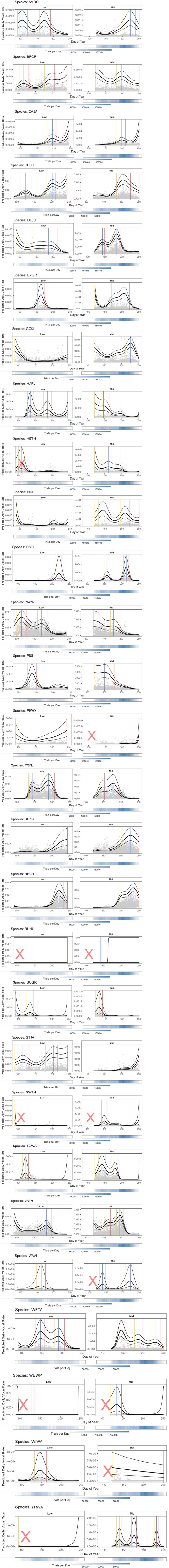
GAM outputs and raw (thresholded) daily success rates for 29 bird species in Olympic National Park. Colored vertical lines correspond to estimated phenometrics (yellow = half‐rises, blue = maxima, red = half‐declines). Translucent dots represent raw (thresholded) daily success rates, scaled to the maximum value of predicted daily vocal rate, for legibility. The heatmap bar at the bottom of the plots depicts the total number of audio samples collected per day. Red “X” denotes a species × elevation combination not included in phenometric analysis because of a lack of range overlap and/or paucity of data.

GAMs flexibly fit curves to detection data, producing a variety of shapes (Appendix, Figure A1). The most common shape of fitted vocal activity was a single peak, exhibited by 47% (11/23) and 59% (13/22) of species in the Low and Mid‐elevation strata, respectively. A bimodal curve was the second most common shape, exhibited by 8 species in each stratum (34%–36%). For these species, on average, the first peak occurred May 20–21 in both elevational strata, whereas the second occurred on 6 July in Low elevations and 16 July in Mid elevations. No peaks were detected for four species in the low‐elevation stratum (Golden‐crowned Kinglet [ *Regulus satrapa* ], Northern Flicker [ *Colaptes auratus* ], Pileated Woodpecker [ *Dryocopus pileatus* ], and Red‐breasted Nuthatch [ *Sitta canadensis* ]). Phenoperiod could not be defined for two species in the Low stratum (Northern Flicker and Red‐breasted Nuthatch) and one species in the Mid‐elevation stratum (Steller's Jay [ *Cyanocitta stelleri* ]) because of start or end dates that were not identifiable by the model.

All but 3 resident species at both elevation strata were discernibly vocal (their vocal activity rate was > 30% of their seasonal maximum rate) at the start of the recording period, April 1, as were two short‐distance migrants, American Robin ( *Turdus migratorius* ), and Dark‐eyed Junco ( *Junco hyemalis* ), at low elevations. In contrast, no long‐distance migrant exhibited discernible increases in vocal activity until 1 May at the earliest (Hammond's Flycatcher [ *Empidonax hammondii* ] in the mid‐elevation stratum).

**FIGURE 3:**
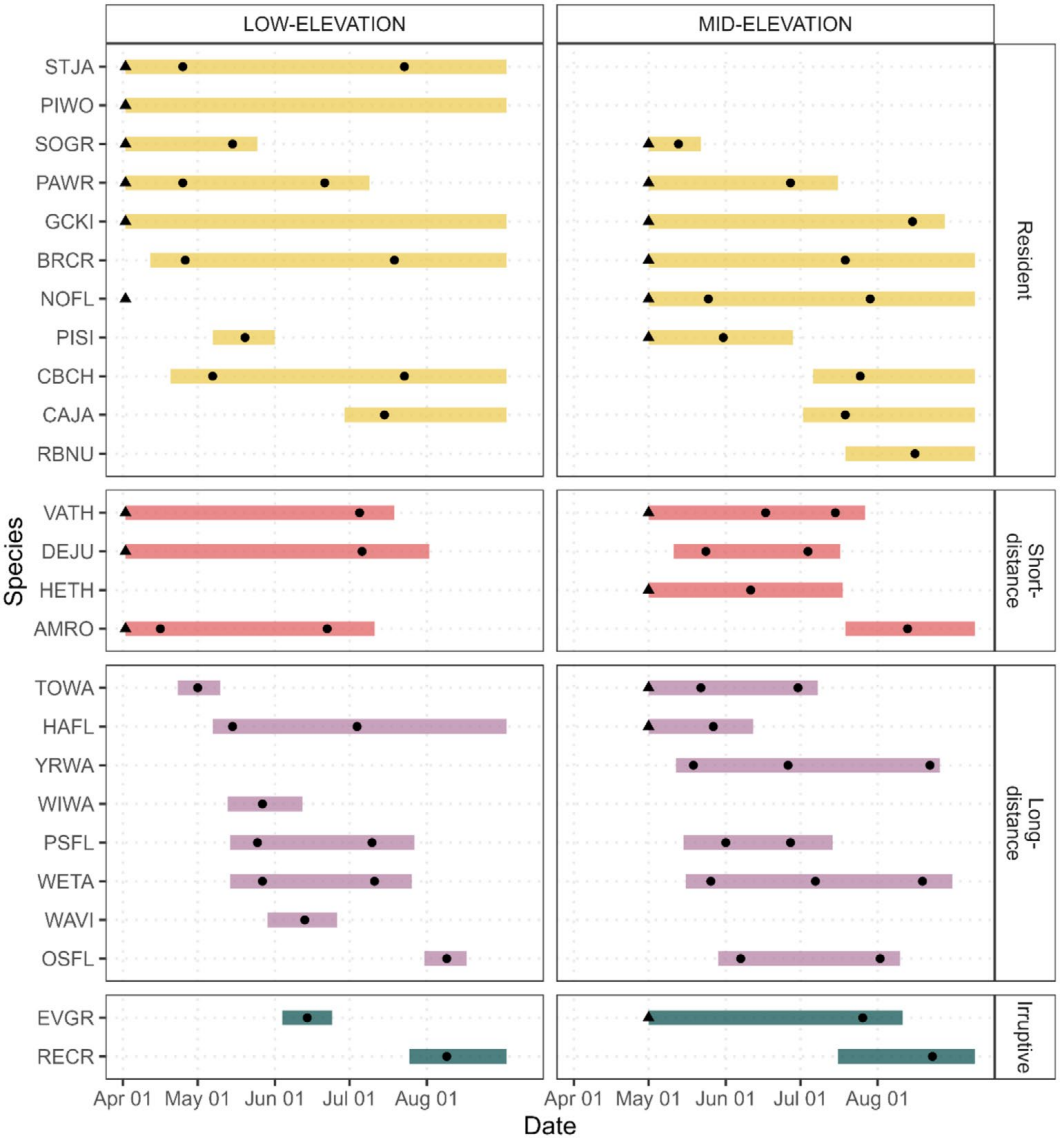
HGAM‐estimated timing and duration of the vocal phenoperiod of 25 common breeding bird species (Table 1) in Olympic National Park. Black dots indicate peaks in vocal activity; black triangles indicate vocal activity within 30% of a local peak at the start of recording. Horizontal bars indicate duration of vocal activity, as calculated by the difference between the last half‐decline (or end boundary) and the first half‐rise (or start boundary).

**FIGURE 4:**
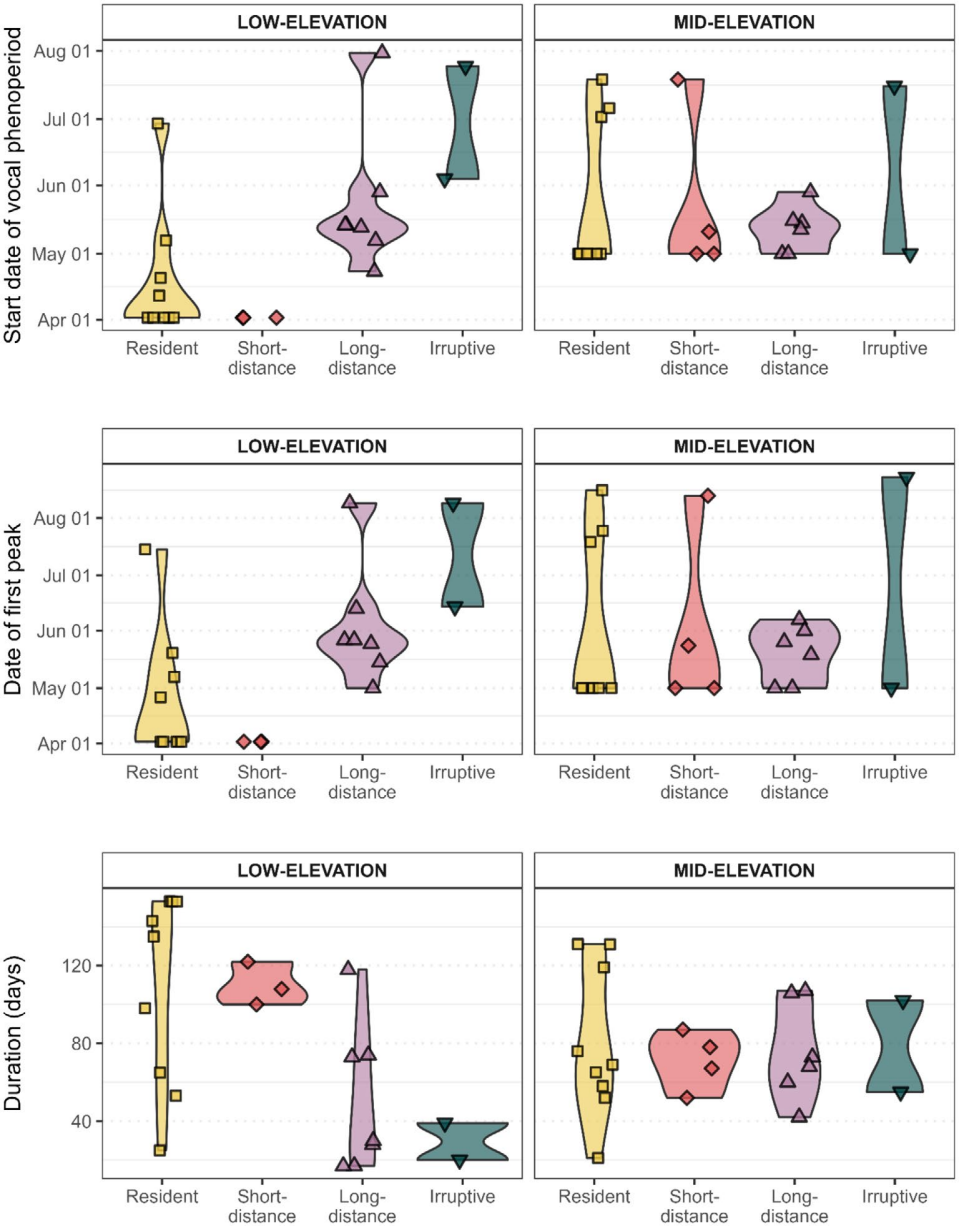
The estimated dates of the onset (top) and peak (middle), as well as the duration (bottom) of vocal phenoperiod in breeding birds in Olympic National Park vary substantially by both elevation and species' migratory strategy.

As measured by the half‐rise, vocal phenoperiods began earlier on average in the Low elevation stratum, and for residents and short‐distance migrants (Figure 3, Figure 4). Within the low‐elevation stratum, the start date varied more between residents and long‐distance migrants, with long‐distance migrants beginning their vocal phenoperiod 34 days later on average than residents (23 May vs. 18 April). In contrast, the start date was more uniform across migratory strategies in the Mid‐elevation stratum—the mean start dates of residents, short‐distance migrants, and long‐distance migrants were all within 12 days of each other (12–24 May). Irruptive species (Evening Grosbeak [ *Coccothraustes vespertinus* ] and Red Crossbill [ *Loxia curvirostra* ]) had later start dates compared to any other group.

Phenoperiod duration was longest in residents, averaging 94 days, moderate in short‐distance migrants (88 days), and shortest in long‐distance migrants (63 days). Elevation influenced both the timing and duration of vocal phenology. In the Mid‐elevation stratum, peak vocal activity within a species lagged by approximately 17 days on average compared to low‐elevation sites, and phenoperiods were typically shorter by 6 days.

Late‐season vocal activity (> 20 July) was prominent in several species, including Red Crossbill, Evening Grosbeak, Chestnut‐backed Chickadee ( *Parus rufescens* ), and Golden‐crowned Kinglet.

## 4 Discussion

A technological revolution in passive detection methods is transforming the study of wildlife ecology, but data streams are outpacing methodological development of appropriate analytical pipelines for end‐user output. We used widely accessible methods, including a passively collected acoustic dataset, an off‐the‐shelf machine‐learning algorithm, and hierarchical generalized additive models, to characterize avian vocal activity as phenological distributions that can be quantified and compared across ecological units of interest. Collecting data on avian breeding phenology typically involves handling wild birds or intensive field efforts; although the insights gained from these high‐effort methods are valuable, even optimal for some study aims, they are constrained in scope. For some questions, passive methods of data collection can minimize impact on birds and increase the capacity to test hypotheses at larger spatial scales and for understudied species. Although PAM is now widely recognized for its capacity to detect species presence and estimate occupancy, its application to phenological questions has been limited by a lack of standardized and ground‐truth workflows. Our methodological pathway here lays the groundwork for using passively collected acoustic data as a broadscale, accessible source of phenological insight.

### 4.1 Phenological Insights

Our first hypothesis concerned the generalizability of the shape of a temperate‐breeding bird's phenoperiod. Nearly all species showed strong seasonality in their vocal output, with half‐rises in vocal activity appearing between 1 May and 1 June for most species. This corresponds to the period encompassing the arrival (for migratory species) and initiation of nesting by most breeding birds in low and mid‐elevation forests of the Pacific Northwest (Ray et al. 2017; Robinson et al. 2019). Increases in song output and breeding‐associated vocalizations (e.g., drumming) likely comprise the bulk of this peak. Song, with its many functions during the breeding period of passerine birds—in territorial arbitration, mate attraction, and pair‐bond strengthening—is arguably one of the most extensively studied topics in bird biology and evolution. Song output, complexity, repertoire size, and detectability have all been documented in many studies of passerine birds as peaking during the early breeding season, and specifically during the periods of nest‐building and egg‐laying (Slagsvold 1977; Lampe and Espmark 1987; Merilä and Sorjonen 1994; Strebel et al. 2014). The ubiquity of peaks in the early breeding season of nearly every species we modeled reflects this well‐documented relationship between birdsong and breeding.

In support of our second and third hypotheses, we found consistent patterns in vocal activity with respect to both migratory strategy and elevation. The vocal phenoperiods of resident birds tended to begin earlier and persist longer than those of migratory or irruptive species. In most cases, residents and some short‐distance migrants were already vocally active at the start of the sampling season (1 April), and some were even at or near a peak in vocal activity, whereas nearly all long‐distance migrants exhibited half‐rises between 1 May and 1 June (Appendix, Figure A1). This result is consistent with the capacity of residents and short‐distance migrants to respond rapidly to favorable local conditions and initiate breeding soon after winter (Newton 2023) as well as phenological differences in food and habitat resources utilized by residents compared to long‐distance migrants (Youngflesh et al. 2023). This pattern also reflects the tendency of residents to utilize vocal signals year‐round for social functions besides breeding (Keating and Reichard 2021; Rose et al. 2022), which may make the half‐rise a less accurate measure of breeding onset specifically for residents than it is for migratory birds when a species‐level classifier is used. Red Crossbill, known for its nomadic tendencies and resource‐tracking behaviors, exhibited peaks in vocal activity in late summer, likely reflecting local recruitment related to the ripening of conifer seeds (Hahn 1995). Elevational delays in phenology—averaging 16 days for peak activity between Low and Mid strata—reflect temperature gradients and phenological delays observed in montane systems (Saracco et al. 2019). These quantifiable shifts across elevations and functional groups provide a foundation for building phenological reaction norms (Inouye et al. 2019; Coe et al. 2021), key to assessing phenological sensitivity to change over environmental or life‐history gradients (Visser et al. 1998; Youngflesh et al. 2023; Tonelli et al. 2024).

### 4.2 Late‐Season Phenology

Many species exhibited second peaks within their vocal phenoperiod. In temperate North America, peaks in vocal activity in late summer are not nearly as well described as breeding‐season song, nor are their functions as well understood. Depending on their timing relative to the first peak, as well as their composition (songs vs. calls), second peaks could be associated with late‐season breeding, with post‐breeding activities, or with both. Second peaks in song output, specifically, have been noted in previous studies focused on singing activity in passerines during the breeding season, but are very rarely studied in their own right (e.g., Greig‐Smith 1982, but see Slagsvold 1977). Such reprises in song late in the breeding season could be linked to renesting attempts after nest failure, singing activity of unpaired males (Merilä and Sorjonen 1994), or onset of second broods in species that clutch doubly (Bruni and Foote 2014). The second vocal peak we observed for Pacific Wren in late June at low elevation, for example, may be comprised at least partly of male song, as they are known to initiate second broods as late as mid‐July (Toews and Irwin 2020). In contrast, second peaks in vocal activity occurring in late July and August are more likely to consist primarily of calls and correspond to post‐breeding phases important to birds' life histories, such as upslope resource tracking and molt‐migration (Boyle and Martin 2015; Wiegardt et al. 2017) or coordination of mixed flocks (Hobson and Wilgenburg 2006). AI classifiers that distinguish vocal classes within species would facilitate the ecological interpretation of these intriguing late‐season peaks.

The final half‐decline of vocal activity is valuable in determining the duration of the vocal phenoperiod. It could also be useful in tracking other post‐breeding activities, such as molt. Seasonal decreases in song output are strongly correlated to gonadal regression, which in many species facilitates the transition out of breeding and into their prebasic molt, an energetically costly period for birds (Dawson 2008) either on their breeding grounds or after performing a molt–migration (Pyle et al. 2018). Acoustic records that extend past the breeding season, paired with fieldwork that correlates acoustic activity with phases of the life cycle such as post‐breeding dispersal, fledgling habitat use and survival, molt, and upslope migration, could facilitate testing of hypotheses about whether these critical but understudied phenophases correlate with vocal phenometrics, and how they relate to survivorship throughout birds' annual cycles.

### 4.3 Directions Forward in the Acoustic Study of Phenology

The field of passive acoustics is rapidly developing in ecology, as sensor technology and AI models converge to unlock new data streams for scientific inference. Our methodological pipeline and hypothesis‐driven exploration of phenological patterns with a case study demonstrate the promise of PAM for future phenological studies in birds and other sound‐producing organisms. However, we uncovered numerous outstanding methodological and ecological questions that currently limit its robustness and utility and deserve research attention. We outline three key areas of development below:

#### 4.3.1 How Well Do Detectable Phenoperiods Correlate With Species' Biology?

The utility of passive monitoring methods for providing insights about phenology and ecology beyond occupancy ultimately depends on how well‐correlated vocal activity signals are with real biological phenomena. One important future direction is thus the ground‐truthing and integration of PAM datasets with field‐collected observations, an approach that would represent a renaissance of the early ornithological studies rooted in natural history and ethology and advance what is known about the complex relationships between vocal activity and behavior in birds. In recent years, scientists have begun to connect vocal activity as measured by PAM to specific phenological events observed in person for a few species, such as with Rock Ptarmigan (Serrurier et al. 2024) and Savannah Sparrow (Moran et al. 2019), but studies like these should be widely replicated in order to understand how vocal phenology and breeding phenology vary over phylogeny, as a function of species' traits (e.g., migratory vs. resident), in relation to different vocalization types, over diel scales (e.g., dawn vocalization vs. mid‐day vs. dusk), over the entire annual cycle, and across broad (e.g., temperate to tropical) and narrow (e.g., elevational) environmental gradients.

#### 4.3.2 Where Will Technical Advancement Improve Inference?

The field of AI models for classification and modeling is developing rapidly, providing many opportunities for additional methodological development at the intersection of deep learning, bioacoustics, and ecology (Xie et al. 2023; van Merriënboer et al. 2024). Increasing accessibility of the tools to efficiently train custom classifiers via transfer learning using the embeddings produced by AI algorithms such as PNW‐Cnet, Perch, HawkEars, and BirdNET (Ruff et al. 2023; Ghani et al. 2023; Huus et al. 2025; Allen‐Ankins et al. 2025) will allow for more efficient development of flexible classifiers (e.g., a classifier specific to a call type, regional dialect, or even individual). Such developments open possibilities for further testing some of the hypotheses we summarize here about birds' vocal phenology.

The ability of a classifier to separate breeding‐specific sounds (e.g., song and drumming) from other vocal classes might provide the greatest leap forward in phenological information content provided by AI classifiers. Quantifying the singing rates of individual birds and tracking them over time (Rognan et al. 2009) or measuring the ratios of vocal signals and/or song types associated with different functions (Trillo and Vehrencamp 2005; Keating and Reichard 2021) would provide key behavioral context for determining whether early‐season vocal activity is attributable to migrating birds, unpaired transients, or birds establishing breeding territories. Similarly, it would provide evidence for whether late‐season vocal activity is attributable to late breeding or post‐breeding activities. Additionally, models that can identify individual birds (or regional dialects) could be instrumental in parsing the difference between vocal activity associated with migratory passage, arrival, or breeding onset. Finally, improving classifier quality for data‐deficient species and in geographic regions where existing pre‐trained classifiers do not perform as well would accommodate more global application of this method. Emerging methods for efficiently training custom models atop pre‐trained classifiers show great promise for improving classifier performance and reducing the time needed to train and verify models (Weldy et al. 2025).

#### 4.3.3 What Biases Present New Challenges for Inference From Acoustic Phenology?

Although potentially unlocking vast data streams on phenology and other ecological indicators, bioacoustic monitoring introduces new biases not present in traditional field data. For example, most PAM analysis methods—including our workflow here—prioritize reducing or eliminating false positives (maximizing classifier “precision”) via thresholding and data verification, at the expense of increasing false negative rates (depressing “recall”). Low classifier performance with respect to precision or recall may skew estimates of any population‐level property being measured, as we demonstrated with our thresholding experiment on Townsend's Warbler. Methods to measure and mitigate the effects of biased classifier behavior on biological inference are in rapid development, and best practices have yet to be established. We highlight two potential sources of bias within this and many PAM workflows below and discuss avenues for addressing them when users replicate this workflow.

Both precision and recall likely vary over time (e.g., as both the stereotypy and composition of bird vocalizations change over a season; Figures S3 and S4) and over space (e.g., with habitat or environmentally mediated sound transmission), which could bias analysis of vocal phenology across these dimensions if left unaccounted for (Navine, Denton, et al. 2024). Preliminary assessment of such “distribution shifts” revealed that BirdNET precision varied seasonally for about one‐quarter of the species we modeled, usually negatively as the season progressed (Table S3). Applying stringent, species‐specific thresholds to the data appeared to mostly (not completely) minimize the effects of poor late‐season precision by eliminating low‐scoring false positives (Figures S5–S8), but revealed to us the importance of more thoroughly investigating how classifier precision varies over environmental context. Further, it is currently unknown how prevalent or problematic distribution shifts in recall are, likely because of how time‐consuming it is to measure. Extremely variable recall across a season may plausibly lead to Type II Error (for example, failure to detect peaks of late‐season vocal activity) and bias ecological conclusions. Work that systematically assesses the variability of recall across time would fill a key knowledge gap in the field of ecoacoustics; without it, a truly complete evaluation of classifier performance is not possible. We encourage users of PAM—whether for studies of occupancy, density, or, like here, phenology—to be wary of raw AI outputs and, especially in the absence of field‐collected data, to identify domains over which distribution shifts could occur (e.g., by species, habitat, time of year, geographic region) and to stratify manual verification efforts of classifier outputs across those domains.

How to optimize validation effort for acoustic datasets and their diverse ecological applications is a major open question in terrestrial bioacoustics (Kitzes et al. 2025). Subjective choices in the method by which subsamples are selected for validation (e.g., number of samples, stratification across scores and other covariates), as well as the choice of threshold itself, may lead to unstable estimates of parameters of interest (Knight and Bayne 2019; Katsis et al. 2025). The subsampling protocol we used for verification (Wood and Kahl 2024) intentionally oversamples BirdNET labels with high (> 0.95) confidence scores in order to more precisely estimate pr(TP) among high‐scoring samples. For some species, this created a highly unbalanced dataset of almost all true positives with which we estimated pr(TP) and potentially led to imprecise threshold estimates. We encourage experimentation on how robust estimates of threshold are to choices in thresholding method and verification effort, in order to further develop best practices. Additionally, the hierarchical model structure we employ here could be flexibly adapted to include direct estimation of false positive rate, as in recent extensions of occupancy models (Rhinehart et al. 2022), or to eliminate thresholding entirely and estimate call density directly within the model using a combination of human‐validated data and the scores themselves (Navine, Camp, et al. 2024; Navine, Denton, et al. 2024).

## 5 Conclusions

PAM holds great potential for understanding complex ecological signals across space, time, and species diversity. Here, we develop a series of analytical methods to extract phenological metrics from PAM and lay out a plausible set of explanations for their ecological and behavioral relevance. We demonstrate these methods using a large case study dataset of bird acoustics in the U.S. Pacific Northwest and test a set of hypotheses that explore how well passive acoustic data can encode signals of phenology across the breeding season of birds. Although we applied this methodological pipeline to a dataset in a temperate region, we encourage its adoption across biomes, study systems, species, and other environmental gradients, ideally paired with field studies to validate the ecological relevance of these patterns in different contexts. Pairing the ever‐widening spatial and temporal reach of PAM with targeted naturalistic field study will both facilitate ground‐truthing and improvement of methods and spur the development and testing of previously unanswerable questions in temporal ecology.

## Author Contributions

**Mary K. Clapp:** conceptualization (equal), data curation (lead), formal analysis (equal), investigation (lead), methodology (equal), software (equal), validation (lead), visualization (lead), writing – original draft (equal), writing – review and editing (lead). **Morgan W. Tingley:** conceptualization (equal), formal analysis (equal), investigation (equal), methodology (equal), software (equal), supervision (equal), writing – original draft (equal), writing – review and editing (equal). **Damon B. Lesmeister:** conceptualization (equal), funding acquisition (equal), methodology (equal), project administration (equal), resources (equal), supervision (equal), writing – review and editing (equal). **Scott A. Gremel:** data curation (equal), funding acquisition (equal), investigation (equal), project administration (equal), resources (equal), supervision (equal), writing – review and editing (equal). **Jason I. Ransom:** conceptualization (equal), funding acquisition (equal), project administration (equal), supervision (equal), writing – review and editing (equal). **Mandy L. Holmgren:** data curation (equal), investigation (equal), validation (equal). **Rodney B. Siegel:** conceptualization (equal), funding acquisition (equal), project administration (equal), supervision (equal), writing – review and editing (equal).

## Funding

This work was supported by the National Park Service (P22AC02338).

## Conflicts of Interest

The authors declare no conflicts of interest.

## Supporting information


**Data S1:** ece373020‐sup‐0001‐supinfo.docx.

## Data Availability

Code and data necessary for replicating the contents of this manuscript are available on the first author's Github page (code, URL: https://github.com/mkclapp/VocalPhenology) and Zenodo (data; https://doi.org/10.5281/zenodo.17644093).

## References

[ece373020-bib-0001] Allen‐Ankins, S. , S. Hoefer , J. Bartholomew , S. Brodie , and L. Schwarzkopf . 2025. “The Use of BirdNET Embeddings as a Fast Solution to Find Novel Sound Classes in Audio Recordings.” Frontiers in Ecology and Evolution 12: 1409407.

[ece373020-bib-0002] Best, L. B. 1981. “Seasonal Changes in Detection of Individual Bird Species.” Studies in Avian Biology 6: 252–261.

[ece373020-bib-0003] Blumenrath, S. H. , and T. Dabelsteen . 2004. “Degradation of Great Tit (*Parus Major*) Song Before and After Foliation: Implications for Vocal Communication in a Deciduous Forest.” Behaviour 141: 935–958.

[ece373020-bib-0004] Boyle, A. W. , B. K. Sandercock , and K. Martin . 2016. “Patterns and Drivers of Intraspecific Variation in Avian Life History Along Elevational Gradients: A Meta‐Analysis.” Biological Reviews 91: 469–482. 25765584 10.1111/brv.12180

[ece373020-bib-0005] Boyle, W. A. , and K. Martin . 2015. “The Conservation Value of High Elevation Habitats to North American Migrant Birds.” Biological Conservation 192: 461–476.

[ece373020-bib-0006] Bruni, A. , and J. R. Foote . 2014. “Dawn Singing of Eastern Phoebes Varies With Breeding Stage and Brood Number.” Wilson Journal of Ornithology 126: 500–507.

[ece373020-bib-0007] Buxton, R. T. , M. F. McKenna , M. Clapp , et al. 2018. “Efficacy of Extracting Indices From Large‐Scale Acoustic Recordings to Monitor Biodiversity.” Conservation Biology 32: 1174–1184. 29676813 10.1111/cobi.13119

[ece373020-bib-0008] Chambert, T. , J. H. Waddle , D. A. W. Miller , S. C. Walls , and J. D. Nichols . 2018. “A New Framework for Analysing Automated Acoustic Species Detection Data: Occupancy Estimation and Optimization of Recordings Post‐Processing.” Methods in Ecology and Evolution 9: 560–570.

[ece373020-bib-0009] Coe, S. J. , K. L. Purcell , and J. T. Rotenberry . 2021. “Modeling Phenological Reaction Norms Over an Elevational Gradient Reveals Contrasting Strategies of Dusky Flycatchers and Mountain Chickadees in Response to Early‐Season Temperatures.” Ornithology 138: ukab056.

[ece373020-bib-0010] Cole, J. S. , N. L. Michel , S. A. Emerson , and R. B. Siegel . 2022. “Automated Bird Sound Classifications of Long‐Duration Recordings Produce Occupancy Model Outputs Similar to Manually Annotated Data.” Ornithological Applications 124: duac003.

[ece373020-bib-0011] Dawson, A. 2008. “Control of the Annual Cycle in Birds: Endocrine Constraints and Plasticity in Response to Ecological Variability.” Philosophical Transactions of the Royal Society, B: Biological Sciences 363: 1621–1633. 10.1098/rstb.2007.0004 PMC2606722 18048294

[ece373020-bib-0012] Furnas, B. J. , and M. C. McGrann . 2018. “Using Occupancy Modeling to Monitor Dates of Peak Vocal Activity for Passerines in California.” Condor 120: 188–200.

[ece373020-bib-0013] Ghani, B. , T. Denton , S. Kahl , and H. Klinck . 2023. “Global Birdsong Embeddings Enable Superior Transfer Learning for Bioacoustic Classification.” Scientific Reports 13: 1–14. 38129622 10.1038/s41598-023-49989-z PMC10739890

[ece373020-bib-0014] Gilbert, W. M. 2022. “Late Breeding Season Definitive Prebasic Molt in Males, and Late Breeding Season Brood Care by Females, in Central California Wilson's Warblers.” Ecology and Evolution 12: e8689. 35342617 10.1002/ece3.8689 PMC8932079

[ece373020-bib-0015] Greig‐Smith, P. W. 1982. “Seasonal Patterns of Song Production by Male Stonechats *Saxicola torquata* .” Ornis Scandinavica 13: 225.

[ece373020-bib-0016] Hahn, T. P. 1995. “Integration of Photoperiodic and Food Cues to Time Changes in Reproductive Physiology by an Opportunistic Breeder, the Red Crossbill, *Loxia Curvirostra* (Aves: Carduelinae).” Journal of Experimental Zoology 272: 213–226.

[ece373020-bib-0017] Hahn, T. P. , K. R. Brazeal , E. M. Schultz , et al. 2015. “Annual Schedules.” In Sturkie's Avian Physiology, 847–867. Elsevier.

[ece373020-bib-0077] Heit, D. R. , W. Ortiz‐Calo , M. K. P. Poisson , A. R. Butler , and R. J. Moll . 2024. “Generalized Nonlinearity in Animal Ecology: Research, Review, and Recommendations.” Ecology and Evolution 14: e11387. 10.1002/ece3.11387. 38994210 PMC11237342

[ece373020-bib-0018] Hobson, K. A. , and S. V. Wilgenburg . 2006. “Composition and Timing of Postbreeding Multispecies Feeding Flocks of Boreal Forest Passerines in Western Canada.” Wilson Journal of Ornithology 118: 164–172.

[ece373020-bib-0019] Huus, J. , K. G. Kelly , E. M. Bayne , and E. C. Knight . 2025. “HawkEars: A Regional, High‐Performance Avian Acoustic Classifier.” Ecological Informatics 87: 103122.

[ece373020-bib-0020] Inouye, B. D. , J. Ehrlén , and N. Underwood . 2019. “Phenology as a Process Rather Than an Event: From Individual Reaction Norms to Community Metrics.” Ecological Monographs 89: e01352.

[ece373020-bib-0021] Kahl, S. , C. M. Wood , M. Eibl , and H. Klinck . 2021. “BirdNET: A Deep Learning Solution for Avian Diversity Monitoring.” Ecological Informatics 61: 101236.

[ece373020-bib-0022] Katsis, L. K. D. , T. A. Rhinehart , E. Dorgay , et al. 2025. “A Comparison of Statistical Methods for Deriving Occupancy Estimates From Machine Learning Outputs.” Scientific Reports 15: 14700. 40289178 10.1038/s41598-025-95207-3 PMC12034756

[ece373020-bib-0023] Keating, H. R. , and D. G. Reichard . 2021. “Seasonal Song Variation in Male Carolina Wrens (*Thryothorus ludovicianus*).” Wilson Journal of Ornithology 133: 365–371.

[ece373020-bib-0024] Kitzes, J. , R. Blake , S. Bombaci , et al. 2021. “Expanding NEON Biodiversity Surveys With New Instrumentation and Machine Learning Approaches.” Ecosphere 12: e03795.

[ece373020-bib-0025] Kitzes, J. , L. Chronister , C. Czarnecki , et al. 2025. “Integrating AI Models Into Ecological Research Workflows: The Case of Terrestrial Bioacoustics.” Methods in Ecology and Evolution: 1–15.

[ece373020-bib-0026] Knight, E. , T. Rhinehart , D. R. de Zwaan , et al. 2024. “Individual Identification in Acoustic Recordings.” Trends in Ecology & Evolution 39, no. 10: 947–960. 38862357 10.1016/j.tree.2024.05.007

[ece373020-bib-0027] Knight, E. C. , and E. M. Bayne . 2019. “Classification Threshold and Training Data Affect the Quality and Utility of Focal Species Data Processed With Automated Audio‐Recognition Software.” Bioacoustics 28: 539–554.

[ece373020-bib-0028] Lampe, H. M. , and Y. O. Espmark . 1987. “Singing Activity and Song Pattern of the Redwing *Turdus iliacus* During the Breeding Season.” Ornis Scandinavica 18: 179.

[ece373020-bib-0029] Lapp, S. , T. Rhinehart , L. Freeland‐Haynes , J. Khilnani , A. Syunkova , and J. Kitzes . 2023. “OpenSoundscape: An Open‐Source Bioacoustics Analysis Package for Python.” Methods in Ecology and Evolution 14: 2321–2328.

[ece373020-bib-0030] Lesmeister, D. B. , and J. M. A. Jenkins . 2022. “Integrating New Technologies to Broaden the Scope of Northern Spotted Owl Monitoring and Linkage With USDA Forest Inventory Data.” Frontiers in Forests and Global Change 5: 966978.

[ece373020-bib-0031] McClintock, B. T. , L. L. Bailey , K. H. Pollock , and T. R. Simons . 2010. “Unmodeled Observation Error Induces Bias When Inferring Patterns and Dynamics of Species Occurrence via Aural Detections.” Ecology 91: 2446–2454. 20836466 10.1890/09-1287.1

[ece373020-bib-0032] McGinn, K. , S. Kahl , M. Z. Peery , H. Klinck , and C. M. Wood . 2023. “Feature Embeddings From the BirdNET Algorithm Provide Insights Into Avian Ecology.” Ecological Informatics 74: 101995.

[ece373020-bib-0033] Merilä, J. , and J. Sorjonen . 1994. “Seasonal and Diurnal Patterns of Singing and Song‐Flight Activity in Bluethroats (*Luscinia Svecica*).” Auk 111: 556–562.

[ece373020-bib-0034] Moran, I. G. , K. C. Lukianchuk , S. M. Doucet , et al. 2019. “Diel and Seasonal Patterns of Variation in the Singing Behaviour of Savannah Sparrows (*Passerculus Sandwichensis*).” Avian Research 10: 26.

[ece373020-bib-0035] Navine, A. K. , R. J. Camp , M. J. Weldy , T. Denton , and P. J. Hart . 2024. “Counting the Chorus: A Bioacoustic Indicator of Population Density.” Ecological Indicators 169: 112930.

[ece373020-bib-0036] Navine, A. K. , T. Denton , M. J. Weldy , and P. J. Hart . 2024. “All Thresholds Barred: Direct Estimation of Call Density in Bioacoustic Data.” Frontiers in Bird Science 3: 1380636.

[ece373020-bib-0037] Newton, I. 2023. The Migration Ecology of Birds. Second ed. Elsevier.

[ece373020-bib-0038] Odom, K. J. , M. L. Hall , K. Riebel , K. E. Omland , and N. E. Langmore . 2014. “Female Song Is Widespread and Ancestral in Songbirds.” Nature Communications 5: 3379. 10.1038/ncomms4379 24594930

[ece373020-bib-0039] Oestreich, W. K. , R. Y. Oliver , M. S. Chapman , M. C. Go , and M. F. McKenna . 2024. “Listening to Animal Behavior to Understand Changing Ecosystems.” Trends in Ecology & Evolution 39: 961–973. 38972787 10.1016/j.tree.2024.06.007

[ece373020-bib-0040] Oliver, R. Y. , D. P. W. Ellis , H. E. Chmura , et al. 2018. “Eavesdropping on the Arctic: Automated Bioacoustics Reveal Dynamics in Songbird Breeding Phenology.” Science Advances 4: eaaq1084. 29938220 10.1126/sciadv.aaq1084 PMC6010323

[ece373020-bib-0078] Pedersen, E. J. , D. L. Miller , G. L. Simpson , and N. Ross . 2019. “Hierarchical Generalized Additive Models in Ecology: An Introduction with Mgcv.” PeerJ 7: e6876. 10.7717/peerj.6876. 31179172 PMC6542350

[ece373020-bib-0041] Pérez‐Granados, C. 2023. “BirdNET: Applications, Performance, Pitfalls and Future Opportunities.” Ibis 165: 1068–1075.

[ece373020-bib-0042] Perrins, C. M. 1970. “The Timing of Birds' Breeding Seasons.” Ibis 112: 242–255.

[ece373020-bib-0043] Pieplow, N. 2019. Peterson Field Guide to Bird Sounds of Western North America. First ed. Houghlin Mifflin Harcourt.

[ece373020-bib-0044] Pijanowski, B. C. , L. J. Villanueva‐Rivera , S. L. Dumyahn , et al. 2011. “Soundscape Ecology: The Science of Sound in the Landscape.” Bioscience 61: 203–216.

[ece373020-bib-0045] Pyle, P. , J. F. Saracco , and D. F. DeSante . 2018. “Evidence of Widespread Movements From Breeding to Molting Grounds by North American Landbirds.” Auk 135: 506–520.

[ece373020-bib-0046] Ray, C. , J. F. Saracco , M. L. Holmgren , et al. 2017. “Recent Stability of Resident and Migratory Landbird Populations in National Parks of the Pacific Northwest.” Ecosphere 8: e01902.

[ece373020-bib-0047] Rhinehart, T. A. , D. Turek , and J. Kitzes . 2022. “A Continuous‐Score Occupancy Model That Incorporates Uncertain Machine Learning Output From Autonomous Biodiversity Surveys.” Methods in Ecology and Evolution 13: 1778–1789.

[ece373020-bib-0048] Robinson, W. D. , C. Partipilo , T. A. Hallman , K. Fairchild , and J. P. Fairchild . 2019. “Idiosyncratic Changes in Spring Arrival Dates of Pacific Northwest Migratory Birds.” PeerJ 7: e7999. 31720118 10.7717/peerj.7999 PMC6842555

[ece373020-bib-0049] Rognan, C. B. , J. M. Szewczak , and M. L. Morrison . 2009. “Vocal Individuality of Great Gray Owls in the Sierra Nevada.” Journal of Wildlife Management 73: 755–760.

[ece373020-bib-0050] Rose, E. M. , N. H. Prior , and G. F. Ball . 2022. “The Singing Question: Re‐Conceptualizing Birdsong.” Biological Reviews 97: 326–342. 34609054 10.1111/brv.12800

[ece373020-bib-0051] Ross, S. R. P.‐J. , D. P. O'Connell , J. L. Deichmann , et al. 2023. “Passive Acoustic Monitoring Provides a Fresh Perspective on Fundamental Ecological Questions.” Functional Ecology 37: 959–975.

[ece373020-bib-0052] Ruff, Z. J. , D. B. Lesmeister , J. M. A. Jenkins , and C. M. Sullivan . 2023. “PNW‐Cnet v4: Automated Species Identification for Passive Acoustic Monitoring.” SoftwareX 23: 101473.

[ece373020-bib-0053] Saracco, J. F. , R. B. Siegel , L. Helton , S. L. Stock , and D. F. DeSante . 2019. “Phenology and Productivity in a Montane Bird Assemblage: Trends and Responses to Elevation and Climate Variation.” Global Change Biology 25: 985–996. 30506620 10.1111/gcb.14538

[ece373020-bib-0054] Serrurier, A. , P. Zdroik , R. Isler , et al. 2024. “Mountain Is Calling—Decrypting the Vocal Phenology of an Alpine Bird Species Using Passive Acoustic Monitoring.” Ibis 166: 1338–1353.

[ece373020-bib-0055] Sethi, S. S. , A. Bick , M.‐Y. Chen , et al. 2024. “Large‐Scale Avian Vocalization Detection Delivers Reliable Global Biodiversity Insights.” Proceedings of the National Academy of Sciences 121: e2315933121.

[ece373020-bib-0056] Siegel, R. B. , R. L. Wilkerson , R. C. Kuntz , J. F. Saracco , and A. L. Holmgren . 2012. “Elevation Ranges of Birds at Mount Rainier National Park, North Cascades National Park Complex, and Olympic National Park, Washington.” Northwestern Naturalist 93: 23–39.

[ece373020-bib-0057] Slagsvold, T. 1977. “Bird Song Activity in Relation to Breeding Cycle, Spring Weather, and Environmental Phenology.” Ornis Scandinavica 8: 197.

[ece373020-bib-0058] Socolar, J. B. , P. N. Epanchin , S. R. Beissinger , and M. W. Tingley . 2017. “Phenological Shifts Conserve Thermal Niches in North American Birds and Reshape Expectations for Climate‐Driven Range Shifts.” Proceedings of the National Academy of Sciences 114: 12976–12981. 10.1073/pnas.1705897114 PMC5724251 29133415

[ece373020-bib-0059] Spiers, A. I. , J. A. Royle , C. L. Torrens , and M. B. Joseph . 2022. “Estimating Species Misclassification with Occupancy Dynamics and Encounter Rates: A Semi‐Supervised, Individual‐Level Approach.” Methods in Ecology and Evolution 13: 1528–1539. 10.1111/2041-210X.13858.

[ece373020-bib-0060] Strebel, N. , M. Kéry , M. Schaub , and H. Schmid . 2014. “Studying Phenology by Flexible Modelling of Seasonal Detectability Peaks.” Methods in Ecology and Evolution 5: 483–490.

[ece373020-bib-0061] Sugai, L. S. M. , T. S. F. Silva , J. W. Ribeiro , and D. Llusia . 2019. “Terrestrial Passive Acoustic Monitoring: Review and Perspectives.” Bioscience 69: 15–25.

[ece373020-bib-0076] Thackeray, S. J. , P. A. Henrys , D. Hemming , et al. 2016. “Phenological Sensitivity to Climate Across Taxa and Trophic Levels.” Nature 535: 241–245. 10.1038/nature18608. 27362222

[ece373020-bib-0062] Toews, D. P. L. , and D. E. Irwin . 2020. Pacific Wren (Troglodytes pacificus), version 1.0. Cornell Lab of Ornithology.

[ece373020-bib-0063] Tonelli, B. A. , C. Youngflesh , T. Cox , M. H. C. Neate‐Clegg , E. B. Cohen , and M. W. Tingley . 2024. “Spatial Nonstationarity in Phenological Responses of Nearctic Birds to Climate Variability.” Ecology Letters 27: e14526. 39374328 10.1111/ele.14526

[ece373020-bib-0064] Tosa, M. I. , E. H. Dziedzic , C. L. Appel , et al. 2021. “The Rapid Rise of Next‐Generation Natural History.” Frontiers in Ecology and Evolution 9: 698131.

[ece373020-bib-0065] Trillo, P. A. , and S. L. Vehrencamp . 2005. “Song Types and Their Structural Features Are Associated With Specific Contexts in the Banded Wren.” Animal Behaviour 70: 921–935. 17173097 10.1016/j.anbehav.2005.02.004 PMC1702368

[ece373020-bib-0066] van Merriënboer, B. , J. Hamer , V. Dumoulin , E. Triantafillou , and T. Denton . 2024. “Birds, Bats and Beyond: Evaluating Generalization in Bioacoustics Models.” Frontiers in Bird Science 3: 1369756.

[ece373020-bib-0067] Visser, M. E. , A. J. van Noordwijk , J. M. Tinbergen , and C. M. Lessells . 1998. “Warmer Springs Lead to Mistimed Reproduction in Great Tits (*Parus major*).” Proceedings of the Royal Society of London, Series B: Biological Sciences 265: 1867–1870.

[ece373020-bib-0068] Weldy, M. J. , D. B. Lesmeister , T. Denton , et al. 2025. “Simulated Soundscapes and Transfer Learning Boost the Performance of Acoustic Classifiers Under Data Scarcity.” Methods in Ecology and Evolution 00: 1–17.

[ece373020-bib-0069] Wiegardt, A. , J. Wolfe , C. J. Ralph , J. L. Stephens , and J. Alexander . 2017. “Postbreeding Elevational Movements of Western Songbirds in Northern California and Southern Oregon.” Ecology and Evolution 7: 7750–7764. 29043031 10.1002/ece3.3326 PMC5632634

[ece373020-bib-0070] Wildlife Acoustics . 2024. “Song Meter SM4 Bioacoustics Recorder User Guide [PDF].” https://www.wildlifeacoustics.com/uploads/user‐guides/SM4‐USER‐GUIDE‐EN‐2024‐06‐11.pdf.

[ece373020-bib-0071] Wiley, R. H. , and D. G. Richards . 1978. “Physical Constraints on Acoustic Communication in the Atmosphere: Implications for the Evolution of Animal Vocalizations.” Behavioral Ecology and Sociobiology 3: 69–94.

[ece373020-bib-0072] Wood, C. M. , and S. Kahl . 2024. “Guidelines for Appropriate Use of BirdNET Scores and Other Detector Outputs.” Journal of Ornithology 165: 777–782.

[ece373020-bib-0079] Wood, S. N. 2017. Generalized Additive Models: An Introduction with R. Second Edition, 2nd ed. Chapman and Hall/CRC. 10.1201/9781315370279.

[ece373020-bib-0073] Wu, J. X. , M. A. Harbison , S. Beilke , P. Saha , and B. L. Bateman . 2025. “A Focus on Females Can Improve Science and Conservation.” Ibis 167: 819–827.

[ece373020-bib-0074] Xie, J. , Y. Zhong , J. Zhang , S. Liu , C. Ding , and A. Triantafyllopoulos . 2023. “A Review of Automatic Recognition Technology for Bird Vocalizations in the Deep Learning Era.” Ecological Informatics 73: 101927.

[ece373020-bib-0075] Youngflesh, C. , G. A. Montgomery , J. F. Saracco , et al. 2023. “Demographic Consequences of Phenological Asynchrony for North American Songbirds.” Proceedings of the National Academy of Sciences 120: e2221961120. 10.1073/pnas.2221961120 PMC10334763 37399376

