# Supplementary material for: Passive Acoustic Data as Phenological Distributions: Uncovering Signals of Temporal Ecology
Source: Ecol Evol. 2026 Feb 3;16(2):e73020. doi: 10.1002/ece3.73020 (PMC12865506; doi:10.1002/ece3.73020)
Supplement: Supplementary file 1 — Data S1: ece373020‐sup‐0001‐supinfo.docx. [file ECE3-16-e73020-s001.docx]

Supplemental Material for “Passive acoustic data as phenological distributions: uncovering signals of temporal ecology”

Table S1. A validated subsample of BirdNET Iabels sampled across confidence scores 0.1-1.0 for each of 29 species and the relative contributions of different vocal classes (e.g., % Song).

| **Common Name** | **Total Samples** | **True Positives (T.P.)** | **Raw Precision (T.P./Total Samples)** | **Song T.P. (# T.P. which are Song)** | **Song T.P./ Total Samples** | **Song T.P./ Total T.P.** |
| --- | --- | --- | --- | --- | --- | --- |
| American Robin | 127 | 123 | 0.97 | 54 | 0.43 | 0.44 |
| Black-throated Gray Warbler | 147 | 49 | 0.33 | 49 | 0.333 | 1.000 |
| Brown Creeper | 200 | 156 | 0.78 | 116 | 0.58 | 0.74 |
| Canada Jay | 195 | 191 | 0.98 | n/a | n/a | n/a |
| Chestnut-backed Chickadee | 200 | 193 | 0.97 | 1 | 0.01 | 0.01 |
| Dark-eyed Junco | 200 | 197 | 0.99 | 140 | 0.7 | 0.71 |
| Evening Grosbeak | 200 | 198 | 0.99 | 1 | 0.01 | 0.01 |
| Golden-crowned Kinglet | 200 | 178 | 0.89 | 62 | 0.31 | 0.35 |
| Hammond's Flycatcher | 200 | 188 | 0.94 | 138 | 0.69 | 0.73 |
| Hermit Thrush | 200 | 195 | 0.98 | 182 | 0.91 | 0.93 |
| Northern Flicker | 200 | 193 | 0.96 | n/a | n/a | n/a |
| Olive-sided Flycatcher | 200 | 197 | 0.99 | 63 | 0.32 | 0.32 |
| Pacific Wren | 200 | 196 | 0.98 | 173 | 0.87 | 0.88 |
| Pileated Woodpecker | 200 | 186 | 0.93 | n/a | n/a | n/a |
| Pine Siskin | 200 | 199 | 1 | 26 | 0.13 | 0.13 |
| Red Crossbill | 200 | 199 | 1 | 1 | 0.01 | 0.01 |
| Red-breasted Nuthatch | 198 | 196 | 0.99 | 78 | 0.39 | 0.4 |
| Rufous Hummingbird | 200 | 105 | 0.53 | n/a | n/a | n/a |
| Sooty Grouse | 200 | 153 | 0.77 | 153 | 0.765 | 1.000 |
| Steller's Jay | 200 | 195 | 0.975 | n/a | n/a | n/a |
| Swainson's Thrush | 200 | 192 | 0.96 | 133 | 0.67 | 0.69 |
| Townsend's Warbler | 200 | 166 | 0.83 | 166 | 0.83 | 1 |
| Varied Thrush | 200 | 185 | 0.93 | 184 | 0.92 | 0.99 |
| Warbling Vireo | 200 | 190 | 0.95 | 134 | 0.67 | 0.71 |
| Western Flycatcher | 200 | 198 | 0.99 | 138 | 0.69 | 0.7 |
| Western Tanager | 200 | 196 | 0.98 | 122 | 0.61 | 0.62 |
| Western Wood-Pewee | 200 | 178 | 0.89 | 19 | 0.1 | 0.11 |
| Wilson's Warbler | 200 | 189 | 0.95 | 186 | 0.93 | 0.98 |
| Yellow-rumped Warbler | 173 | 122 | 0.71 | 114 | 0.66 | 0.93 |

Table S2. Model output for each species x elevation stratum combination. EDF = Effective degrees of freedom, RDF = Reference degrees of freedom. Deviance explained represents the proportion of the deviance explained by the model and is preferred over R-squared for models with non-Gaussian response variables. “ntrials” = number of trials (3-second windows within which BirdNET made a label); “nhits” = number of BirdNET hits above 95% true-positive probability threshold. Italicized rows indicate models whose outputs were not used in subsequent phenometric analyses.

|  |  | **s(Jday)** | | | **s(location)** | | |  |  |  |
| --- | --- | --- | --- | --- | --- | --- | --- | --- | --- | --- |
| **Species Code** | **Elev. Bin** | **EDF** | **RDF** | **p-value** | **EDF** | **RDF** | **p-value** | **Deviance explained** | **ntrials** | **nhits** |
| AMRO | Low | 5.93 | 6.00 | 0.00 | 59.19 | 64 | 0.00 | 0.326 | 2695 | 6518 |
| AMRO | Mid | 5.77 | 5.98 | 0.00 | 106.75 | 119 | 0.00 | 0.41 | 5043 | 4225 |
| BRCR | Low | 5.90 | 6.00 | 0.00 | 58.73 | 64 | 0.00 | 0.366 | 2695 | 4857 |
| BRCR | Mid | 5.84 | 5.99 | 0.00 | 114.76 | 119 | 0.00 | 0.44 | 5043 | 15344 |
| CAJA | Low | 5.80 | 5.98 | 0.00 | 52.29 | 64 | 0.00 | 0.503 | 2695 | 2540 |
| CAJA | Mid | 5.91 | 6.00 | 0.00 | 106.50 | 119 | 0.00 | 0.435 | 5043 | 3260 |
| CBCH | Low | 6.00 | 6.00 | 0.00 | 63.34 | 64 | 0.00 | 0.497 | 2695 | 192854 |
| CBCH | Mid | 5.99 | 6.00 | 0.00 | 118.12 | 119 | 0.00 | 0.455 | 5043 | 167079 |
| DEJU | Low | 5.94 | 6.00 | 0.00 | 63.41 | 64 | 0.00 | 0.558 | 2695 | 56230 |
| DEJU | Mid | 6.00 | 6.00 | 0.00 | 118.56 | 119 | 0.00 | 0.571 | 5043 | 161508 |
| EVGR | Low | 5.97 | 6.00 | 0.00 | 57.26 | 64 | 0.00 | 0.716 | 2695 | 5464 |
| EVGR | Mid | 5.97 | 6.00 | 0.00 | 113.75 | 119 | 0.00 | 0.434 | 5043 | 12328 |
| GCKI | Low | 5.91 | 6.00 | 0.00 | 60.63 | 64 | 0.00 | 0.469 | 2695 | 10235 |
| GCKI | Mid | 5.99 | 6.00 | 0.00 | 117.65 | 119 | 0.00 | 0.41 | 5043 | 58416 |
| HAFL | Low | 6.00 | 6.00 | 0.00 | 59.16 | 64 | 0.00 | 0.806 | 2695 | 24027 |
| HAFL | Mid | 6.00 | 6.00 | 0.00 | 113.60 | 119 | 0.00 | 0.537 | 5043 | 20000 |
| *HETH* | *Low* | *5.54* | *5.90* | *0.00* | *35.94* | *64* | *0.00* | *0.493* | *2695* | *158* |
| HETH | Mid | 6.00 | 6.00 | 0.00 | 107.25 | 119 | 0.00 | 0.758 | 5043 | 32102 |
| NOFL | Low | 5.98 | 6.00 | 0.00 | 55.94 | 64 | 0.00 | 0.523 | 2695 | 3234 |
| NOFL | Mid | 5.95 | 6.00 | 0.00 | 111.42 | 119 | 0.00 | 0.463 | 5043 | 14270 |
| OSFL | Low | 5.77 | 5.96 | 0.00 | 44.68 | 64 | 0.00 | 0.832 | 2695 | 3940 |
| OSFL | Mid | 5.99 | 6.00 | 0.00 | 106.65 | 119 | 0.00 | 0.669 | 5043 | 30211 |
| PAWR | Low | 6.00 | 6.00 | 0.00 | 63.93 | 64 | 0.00 | 0.6 | 2695 | 719694 |
| PAWR | Mid | 5.99 | 6.00 | 0.00 | 117.83 | 119 | 0.00 | 0.725 | 5043 | 308118 |
| PISI | Low | 5.95 | 6.00 | 0.00 | 59.34 | 64 | 0.00 | 0.64 | 2695 | 3458 |
| PISI | Mid | 5.93 | 6.00 | 0.00 | 117.08 | 119 | 0.00 | 0.658 | 5043 | 58393 |
| PIWO | Low | 5.07 | 5.67 | 0.00 | 57.43 | 64 | 0.00 | 0.427 | 2695 | 6001 |
| *PIWO* | *Mid* | *5.93* | *6.00* | *0.00* | *83.85* | *119* | *0.00* | *0.549* | *5043* | *1222* |
| PSFL | Low | 6.00 | 6.00 | 0.00 | 63.21 | 64 | 0.00 | 0.709 | 2695 | 717182 |
| PSFL | Mid | 6.00 | 6.00 | 0.00 | 118.24 | 119 | 0.00 | 0.779 | 5043 | 605992 |
| RBNU | Low | 6.00 | 6.00 | 0.00 | 61.88 | 64 | 0.00 | 0.607 | 2695 | 115180 |
| RBNU | Mid | 6.00 | 6.00 | 0.00 | 117.94 | 119 | 0.00 | 0.581 | 5043 | 545420 |
| RECR | Low | 5.99 | 6.00 | 0.00 | 60.00 | 64 | 0.00 | 0.854 | 2695 | 139821 |
| RECR | Mid | 6.00 | 6.00 | 0.00 | 113.89 | 119 | 0.00 | 0.804 | 5043 | 377211 |
| *RUHU* | *Low* | *1.00* | *1.00* | *1.00* | *0.00* | *64* | *1.00* | *NA* | *2695* | *0* |
| *RUHU* | *Mid* | *1.88* | *2.07* | *0.87* | *6.53* | *119* | *0.40* | *0.802* | *5043* | *4* |
| SOGR | Low | 5.69 | 5.91 | 0.00 | 39.65 | 64 | 0.00 | 0.575 | 2695 | 1776 |
| SOGR | Mid | 5.99 | 6.00 | 0.00 | 77.35 | 119 | 0.00 | 0.731 | 5043 | 13915 |
| STJA | Low | 5.97 | 6.00 | 0.00 | 62.39 | 64 | 0.00 | 0.364 | 2695 | 49870 |
| STJA | Mid | 5.96 | 6.00 | 0.00 | 113.09 | 119 | 0.00 | 0.474 | 5043 | 19569 |
| *SWTH* | *Low* | *5.98* | *6.00* | *0.00* | *50.71* | *64* | *0.00* | *0.698* | *2695* | *4594* |
| *SWTH* | *Mid* | *5.81* | *5.98* | *0.00* | *70.93* | *119* | *0.00* | *0.474* | *5043* | *254* |
| TOWA | Low | 5.05 | 5.31 | 0.00 | 34.38 | 64 | 0.00 | 0.816 | 2695 | 1126 |
| TOWA | Mid | 5.99 | 6.00 | 0.00 | 99.09 | 119 | 0.00 | 0.831 | 5043 | 45481 |
| VATH | Low | 6.00 | 6.00 | 0.00 | 63.39 | 64 | 0.00 | 0.555 | 2695 | 168498 |
| VATH | Mid | 6.00 | 6.00 | 0.00 | 118.67 | 119 | 0.00 | 0.619 | 5043 | 270886 |
| WAVI | Low | 5.16 | 5.55 | 0.00 | 28.88 | 64 | 0.00 | 0.821 | 2695 | 1969 |
| *WAVI* | *Mid* | *5.87* | *5.99* | *0.00* | *62.86* | *119* | *0.00* | *0.575* | *5043* | *973* |
| WETA | Low | 5.97 | 6.00 | 0.00 | 60.34 | 64 | 0.00 | 0.427 | 2695 | 15140 |
| WETA | Mid | 5.98 | 6.00 | 0.00 | 110.05 | 119 | 0.00 | 0.639 | 5043 | 11178 |
| *WEWP* | *Low* | *5.47* | *5.74* | *0.00* | *21.68* | *64* | *0.00* | *0.668* | *2695* | *263* |
| *WEWP* | *Mid* | *4.69* | *5.28* | *0.00* | *43.93* | *119* | *0.00* | *0.641* | *5043* | *546* |
| WIWA | Low | 5.93 | 6.00 | 0.00 | 53.88 | 64 | 0.00 | 0.647 | 2695 | 5214 |
| *WIWA* | *Mid* | *1.00* | *1.00* | *0.02* | *81.05* | *119* | *0.00* | *0.766* | *5043* | *2832* |
| *YRWA* | *Low* | *1.88* | *2.34* | *0.28* | *9.64* | *64* | *0.01* | *0.383* | *2695* | *7* |
| YRWA | Mid | 5.95 | 6.00 | 0.00 | 62.56 | 119 | 0.00 | 0.89 | 5043 | 2143 |

Table S3. Results of binomial logistic regression model comparison using Akaike’s Information Criterion (AIC) to assess the prevalence of variable BirdNET classifier precision across season and elevation. “P-val: score” refers to the p-value associated with the z-statistic for the *score* parameter in the score-only (base) model. In contrast, p-values for *Jday* and *ElevBin* (columns 3-4) refer to the results of a Chi-square test assessing whether the second model provides a significantly better fit than the first by including the additional parameter. Columns “n(Low)” and “n(Mid)” refer to the number of verification samples from either elevation stratum. We considered the best model the base model unless another model’s AIC was lower by ΔAIC > 3. Asterisks in the “best model” column refer to species for which vocal phenology was not modeled for both elevation strata due to lack of range overlap and/or a paucity of data.

| **Dataset** | **p-val: score** | **p-val: +JDay** | **p-val: +ElevBin** | **n(Low)** | **n(Mid)** | **AIC (*score* model)** | **AIC (*score + jday* model)** | **AIC (*score + elevbin* model)** | **best model** |
| --- | --- | --- | --- | --- | --- | --- | --- | --- | --- |
| AMRO | 0.244 | 0.091 | 0.003 | 81 | 44 | 35.8269 | 34.97561 | 28.73461 | elev |
| BRCR | 0.000 | 0.478 | 0.089 | 53 | 138 | 125.062 | 126.5576 | 124.1679 | base |
| BTYW | 0.000 | 0.307 | 0.735 | 32 | 113 | 172.464 | 173.4221 | 174.3498 | base |
| CAJA | 0.067 | 0.171 | 0.468 | 74 | 116 | 39.59451 | 39.72301 | 41.06882 | base |
| CBCH | 0.024 | 0.866 | 0.173 | 116 | 81 | 48.43563 | 50.40696 | 48.58279 | base |
| DEJU | 0.244 | 0.710 | 0.220 | 55 | 132 | 29.88243 | 31.74414 | 30.38009 | base |
| EVGR | 0.173 | 0.073 | 0.627 | 70 | 125 | 21.25464 | 20.04824 | 23.01894 | base |
| GCKI | 0.000 | 0.246 | 0.660 | 38 | 149 | 116.1446 | 116.7971 | 117.9507 | base |
| HAFL | 0.001 | 0.020 | 0.620 | 97 | 101 | 68.39156 | 64.97117 | 70.1457 | jday |
| HETH | 0.032 | 0.945 | 0.024 | 1 | 181 | 38.02542 | 40.02062 | 34.93132 | elev* |
| NOFL | 0.005 | 0.018 | 0.728 | 49 | 145 | 34.24438 | 30.63679 | 36.12305 | jday |
| OSFL | 0.071 | 0.181 | 0.406 | 14 | 115 | 22.06774 | 22.27634 | 23.37861 | base |
| PAWR | 0.022 | 0.716 | 0.987 | 144 | 53 | 31.78373 | 33.6513 | 33.78348 | base |
| PISI | 0.317 | 0.585 | 0.033 | 7 | 174 | 14.04884 | 15.75018 | 11.48822 | base |
| PIWO | 0.000 | 0.025 | 0.003 | 161 | 39 | 75.90518 | 72.88088 | 69.27206 | elev* |
| PSFL | 0.150 | 0.372 | 0.951 | 89 | 111 | 16.4784 | 17.6825 | 18.47459 | base |
| RBNU | 0.391 | 0.377 | 0.395 | 24 | 159 | 25.29703 | 26.51549 | 26.57278 | base |
| RECR | 0.376 | 0.063 | 0.577 | 42 | 158 | 13.8245 | 12.37877 | 15.51269 | base |
| RUHU | 0.000 | 0.212 | 0.912 | 23 | 168 | 192.771 | 193.2145 | 194.7588 | base |
| SOGR | 0.000 | 0.000 | 0.013 | 22 | 176 | 81.6137 | 61.42813 | 77.48666 | jday |
| STJA | 0.113 | 0.013 | 0.520 | 141 | 57 | 22.39349 | 18.17376 | 23.97944 | jday |
| SWTH | 0.026 | 0.396 | 0.000 | 181 | 18 | 43.94775 | 45.22759 | 20.13651 | elev* |
| TOWA | 0.000 | 0.000 | 0.835 | 15 | 185 | 124.7721 | 98.11859 | 126.7286 | jday |
| VATH | 0.001 | 0.483 | 0.698 | 68 | 126 | 66.31163 | 67.81902 | 68.16109 | base |
| WAVI | 0.004 | 0.066 | 0.005 | 122 | 78 | 68.83323 | 67.46352 | 63.02097 | elev* |
| WETA | 0.122 | 0.353 | 0.571 | 89 | 110 | 35.44512 | 36.58403 | 37.12428 | base |
| WEWP | 0.001 | 0.131 | 0.913 | 35 | 157 | 80.30471 | 80.02509 | 82.29286 | base |
| WIWA | 0.013 | 0.000 | 0.326 | 142 | 58 | 81.4175 | 50.31149 | 82.45098 | jday |
| YRWA | 0.000 | 0.000 | 0.001 | 8 | 160 | 101.2658 | 88.68202 | 91.53028 | jday* |


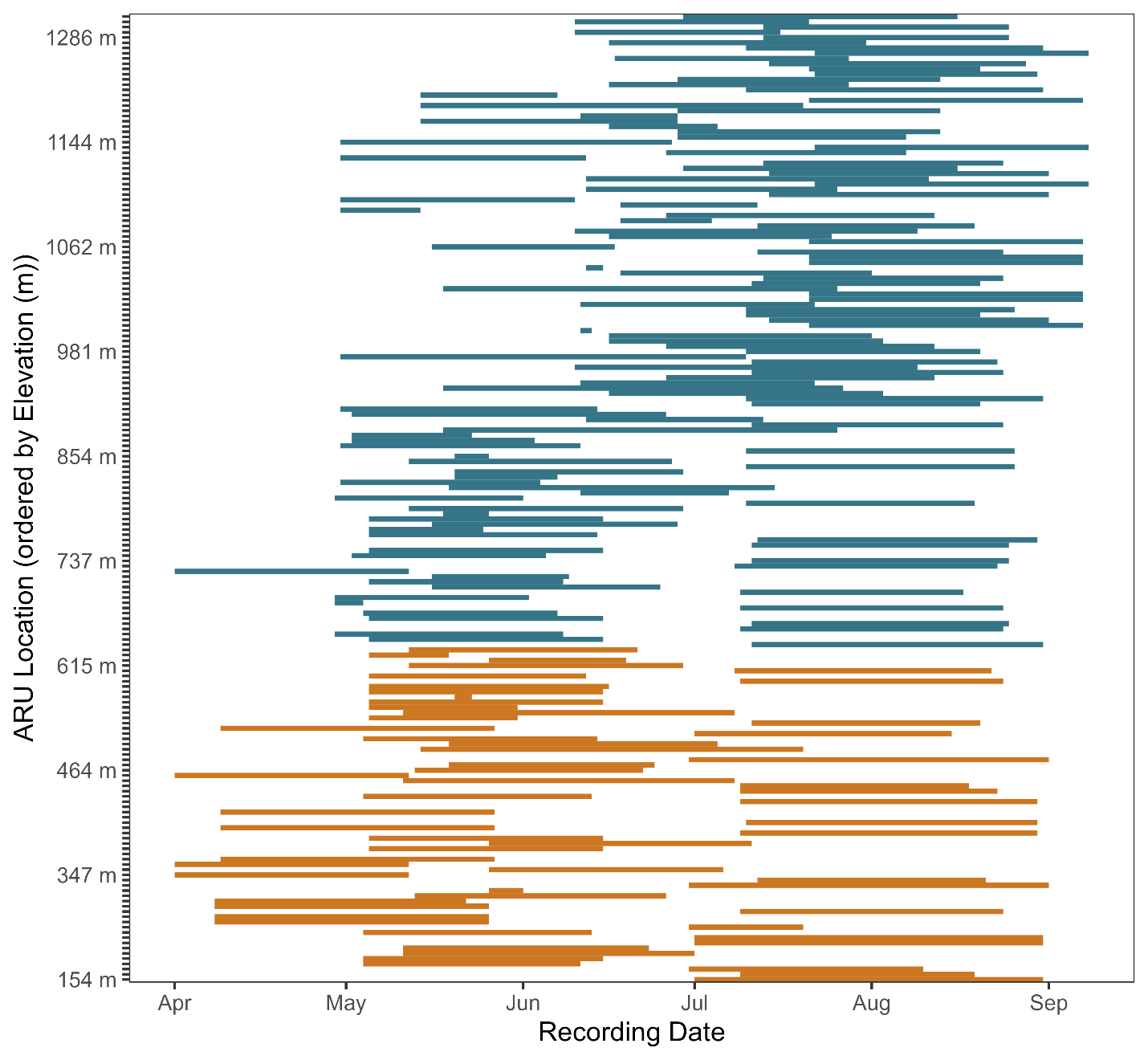
Figure S1. ARU survey effort at Olympic National Park in 2021 ordered by elevation; orange refers to locations within the Low elevation stratum; teal refers to locations within the Mid-elevation stratum. (n = 185 recorders)


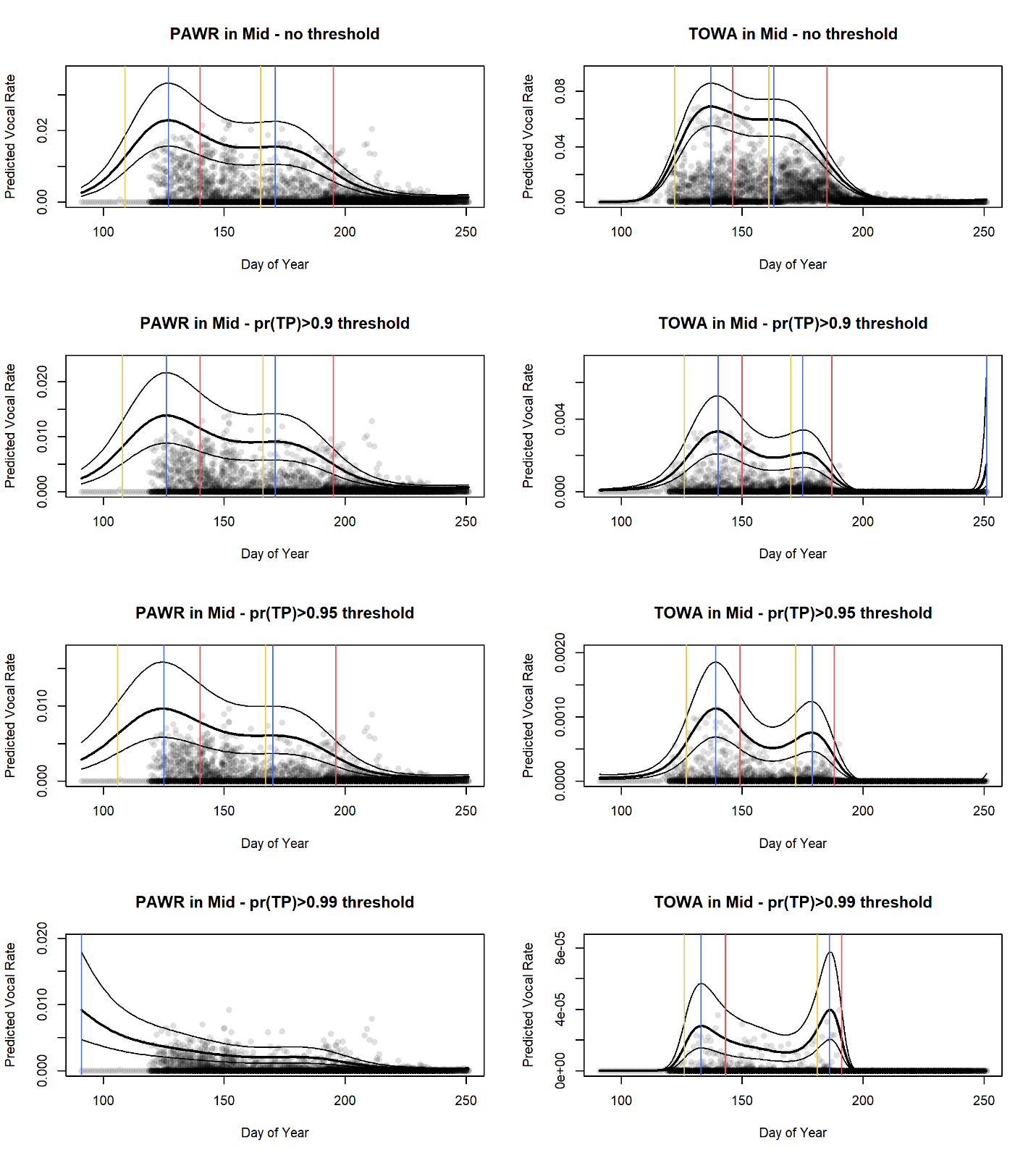


Figure S2. For the Pacific Wren, vocal activity displays the same seasonal patterns regardless of threshold, except for at the highest threshold. For the Townsend's Warbler, seasonal patterns of vocal activity appeared sensitive to threshold, as peaks and troughs of vocal activity appear to shift and/or dissolve with increasing stringency. For example, the date of the second peak (marked in blue) shifted up to 23 days between thresholds, while the second peak for Pacific Wren only shifted by 1 day. Green lines indicate half-rises and half-declines. Blue lines indicate peaks. Points represent daily per-site “success rates” in the raw (thresholded) data, but scaled to the maximum value of the **predicted** vocal rate for each species for easier visualization.


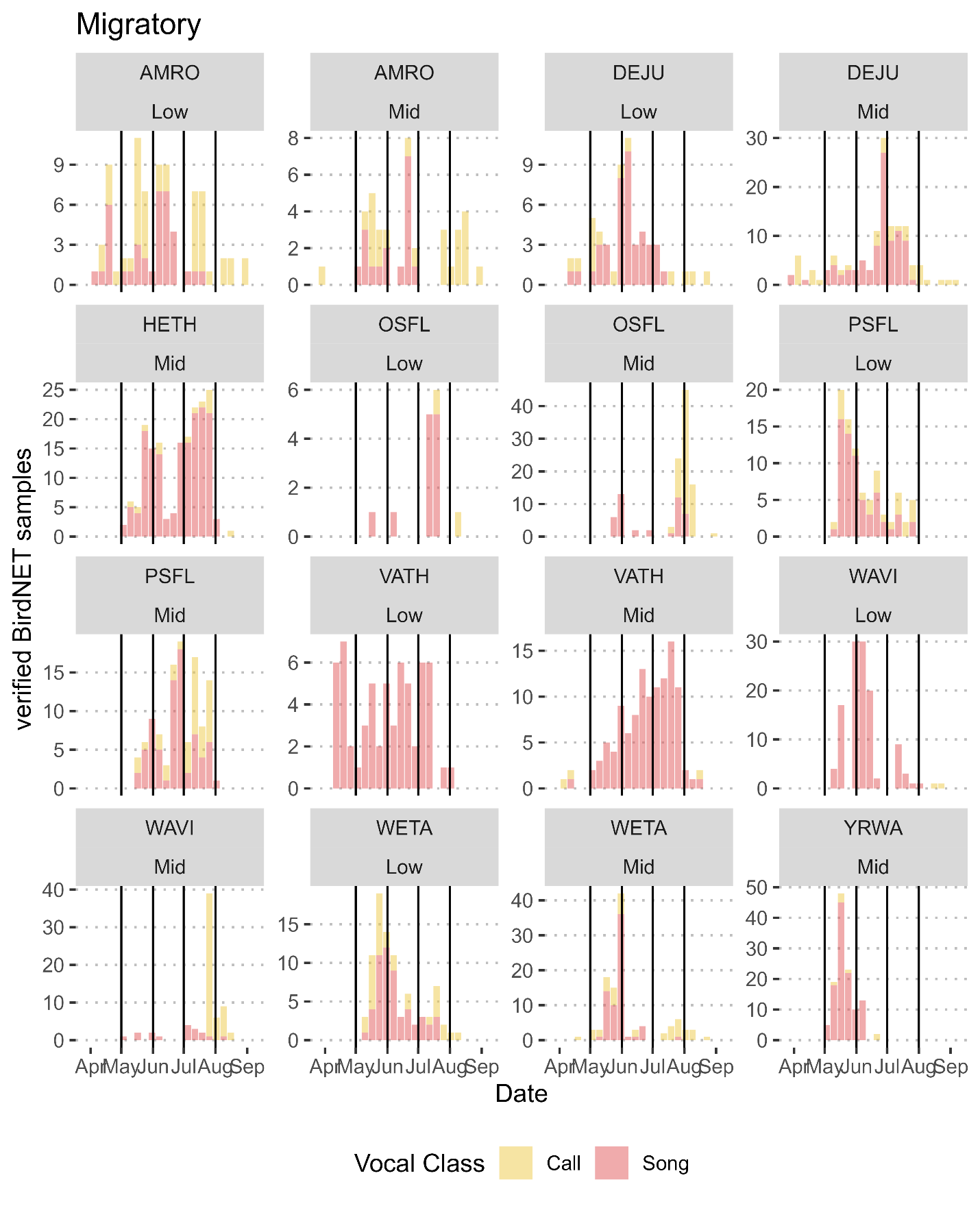


Figure S3. Relative contributions of songs versus calls in migratory birds at Low and Mid elevations over the recording period (April-August) in expert-validated, randomly selected 3-sec clips from an acoustic dataset from Olympic National Park.


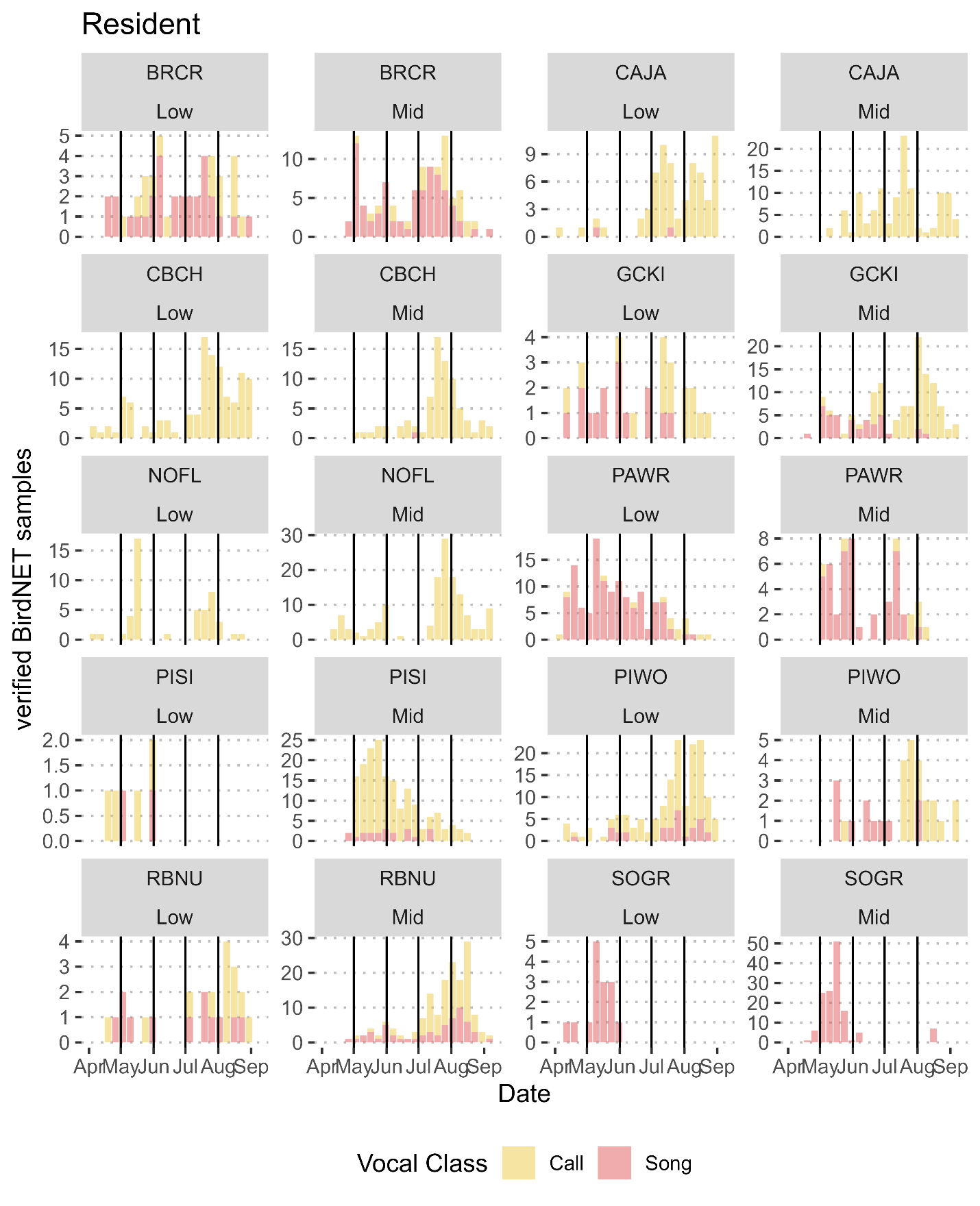


Figure S4. Relative contributions of songs versus calls in resident birds at Low and Mid elevations over the recording period (April-August) in expert-validated, randomly selected 3-sec clips from an acoustic dataset from Olympic National Park.


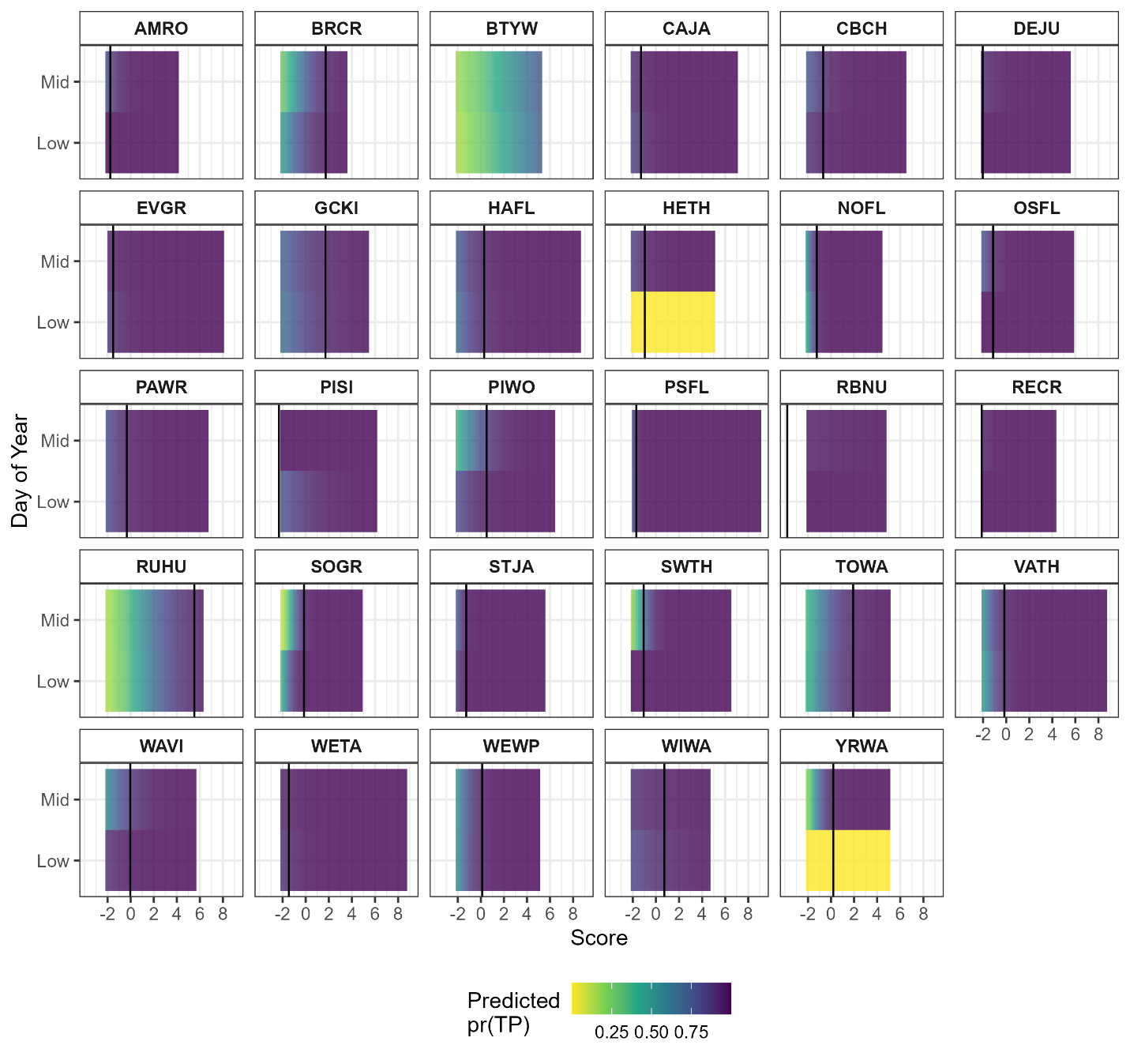


Figure S5. The predicted probability of a True Positive [pr(TP)] across BirdNET scores (on the logit scale) using a binomial logistic regression that includes both score and Elevation Bin varies by species. Vertical black lines indicate the score at which pr(TP) ≥ 0.95 is achieved, calculated by the base (score-only) model used to threshold the BirdNET data for this paper. Please refer to Table S3 for information about which species x Elevation stratum combinations were not modeled due to lack of range overlap and/or paucity of data (e.g., HETH and YRWA in Low).


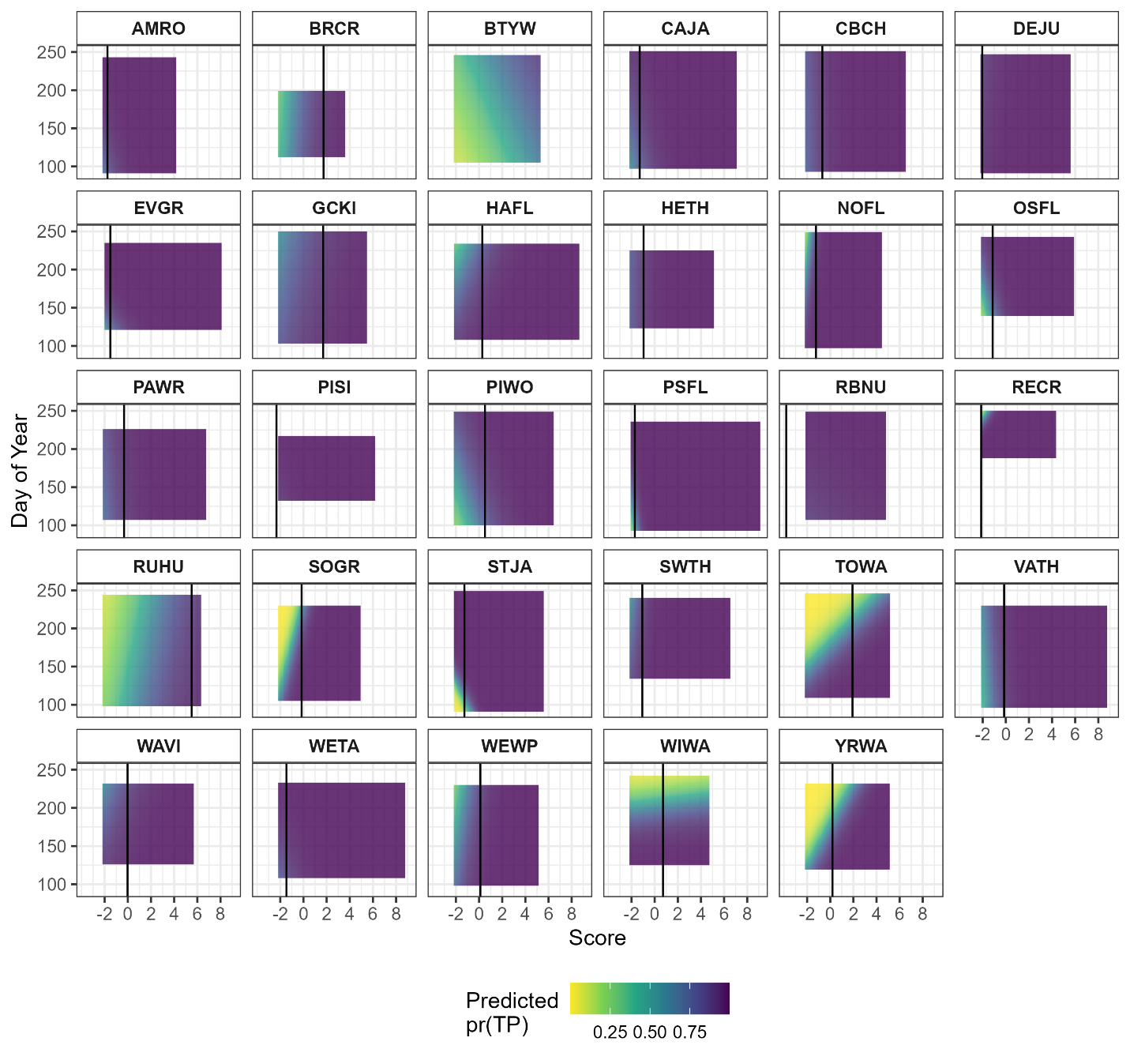
Figure S6. The predicted probability of a True Positive [pr(TP)] across BirdNET scores (on the logit scale) using a binomial logistic regression that includes both score and ordinal day of year varies by species. Vertical black lines indicate the score at which pr(TP) ≥ 0.95 is achieved, calculated by the base (score-only) model used to threshold the BirdNET data for this paper. In most cases, seasonal variability in pr(TP) occurs primarily at very low scores which are thresholded from the data, with the exceptions of TOWA, WIWA, and YRWA, where thresholding does not completely remove scores with pr(TP) < 0.95.


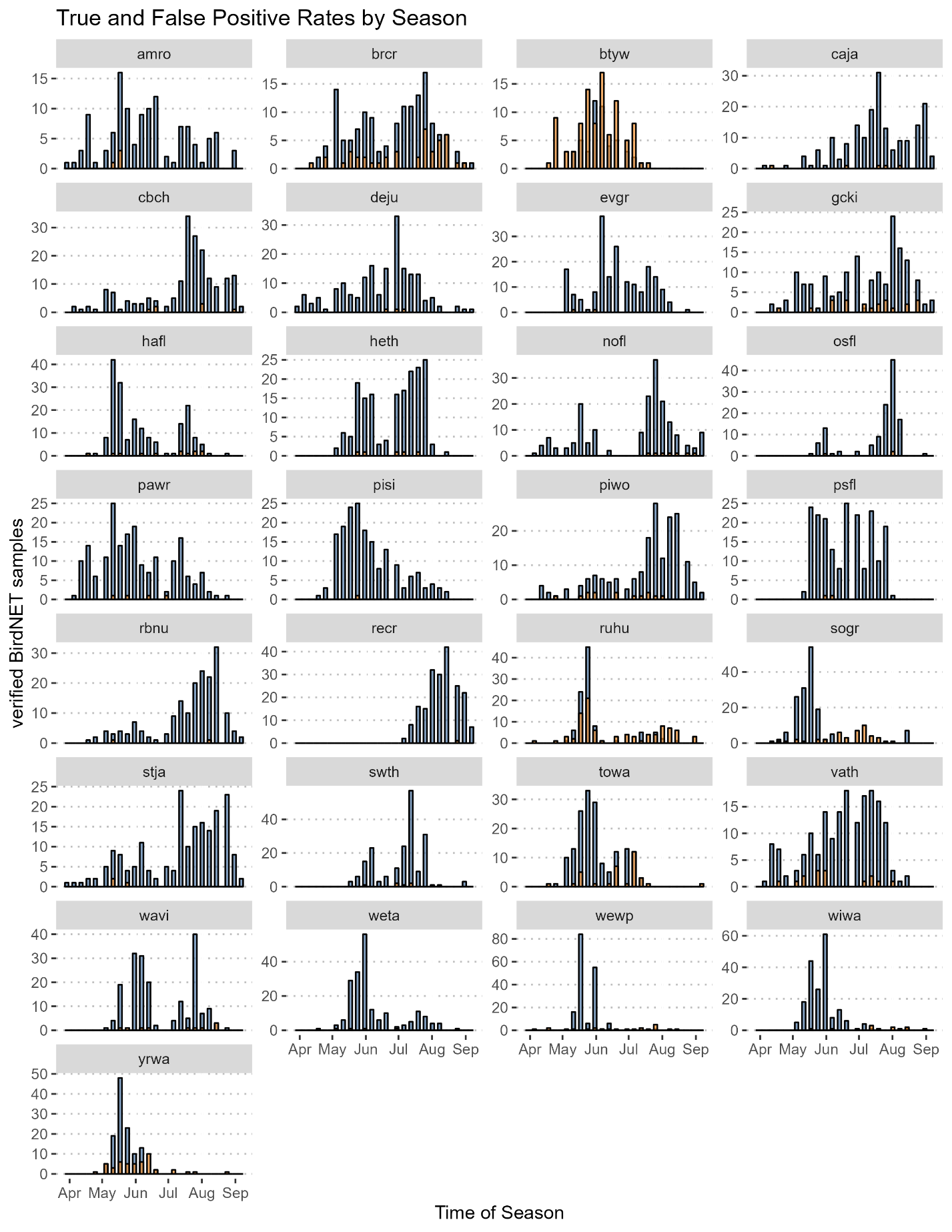


Figure S7. Distribution of unthresholded, verified subsample of true (blue) and false (orange) positive BirdNET labels across the sampling season.


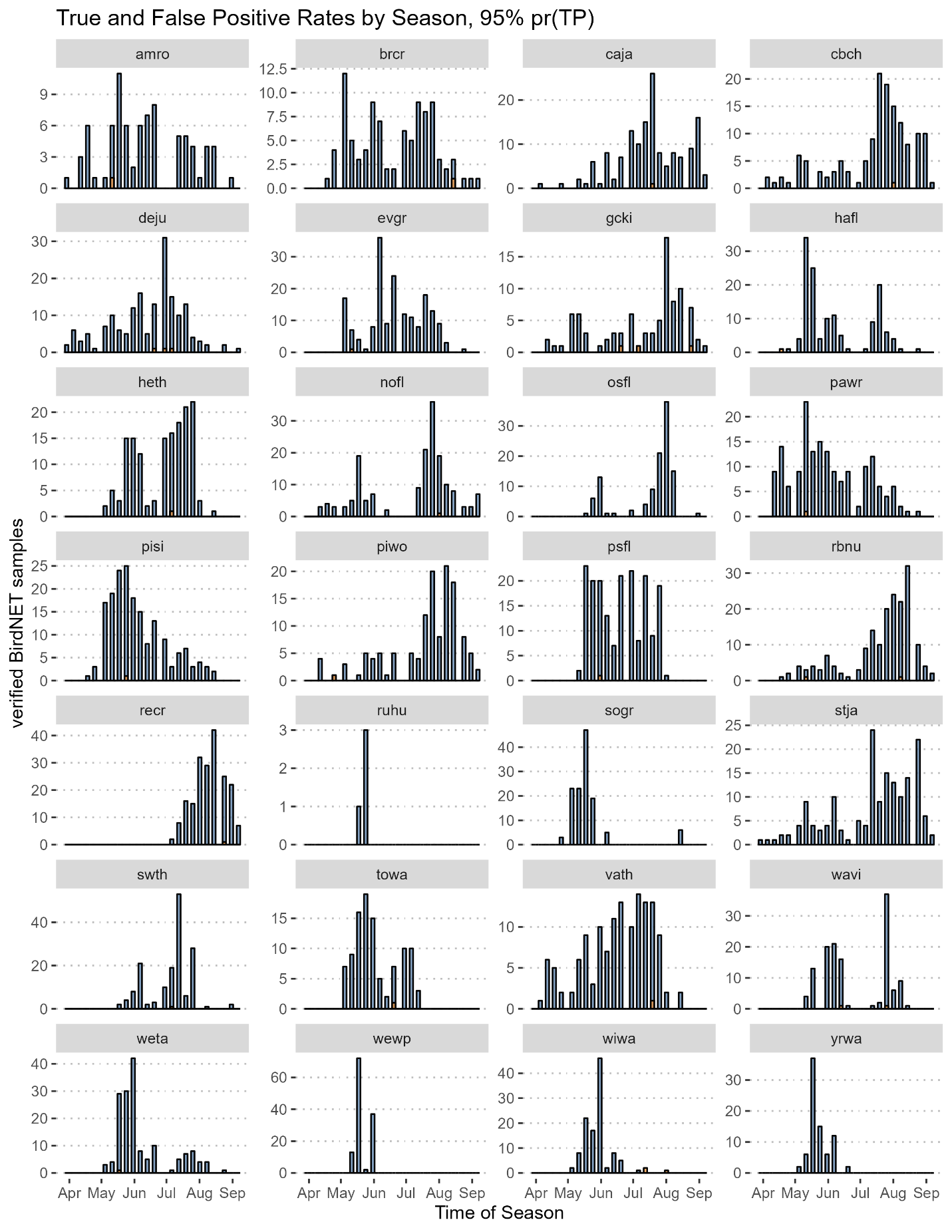
Figure S8. Distribution of verified subsample of true (blue) and false (orange) positive BirdNET labels across the sampling season, with labels with a true-positive probability [pr(TP)] < 0.95 filtered out.
